# Taxonova: Deep learning enables fast and confidence-aware microbiome species-level amplicon classification

**DOI:** 10.1371/journal.pcbi.1014912

**Published:** 2026-10-29

**Authors:** Kardokh Kakabra, Shriram Patel, Andriy Temko, Marcus J. Claesson

**Affiliations:** 1 School of Microbiology, University College Cork, Cork, Ireland; 2 APC Microbiome Ireland, University College Cork, Cork, Ireland; 3 Seqbiome Ltd., Cork, Ireland; 4 Department of Electrical and Electronic Engineering, University College Cork, Cork, Ireland; University of Amsterdam: Universiteit van Amsterdam, NETHERLANDS, KINGDOM OF THE

## Abstract

Accurate species-level taxonomic classification of microbial amplicon sequences remains challenging due to the difficulty of resolving subtle variations within conserved ribosomal regions. Traditional similarity-based methods struggle with short amplicons and sequences with poor database representation. No existing method performs robustly across the full spectrum of amplicon lengths, limiting applicability across diverse sequencing platforms and variable-region targets. We present Taxonova, a deep learning framework for both short- and long-read ribosomal amplicons. Evaluated across physical and biome-simulated mock communities representing seven biomes (via the accompanying benchmark tool Taxobench), Taxonova attains species-level F-measures of 0.76–1.00 (median 0.94) for short-read and 0.82–0.99 (median 0.97) for long-read amplicons spanning 250 bp to 4,900 bp, reduces the species-level abundance-estimation error (L1 norm) by a median of 0.52 to 1.50 depending on amplicon region, and has a species-level false-positive rate that is a median of 16.2 × 10^−4^ lower than that of the other classifiers. Species-level assignment is expected to be most reliable for well-characterised communities, such as human-associated microbiomes. To demonstrate real-world applicability, we also compared amplicon-derived taxonomic profiles to paired shotgun whole-genome sequencing from gut, soil and vaginal microbiomes; Taxonova achieves the strongest species-level concordance (Jaccard) in all three studies, with a species-level Jaccard index 0.009 to 0.022 higher than that of the next-best classifier across both high- and low-complexity communities. Taxonova runs at a throughput of roughly 100 sequences per second on a single CPU core, making it suitable for routine deployment in research settings.

## Author summary

We developed Taxonova to address a long-standing problem in microbiome research: extracting species-level information from short DNA marker sequences. Taxonova abstains when the amplicon region carries too little signal to separate close relatives, returning a genus-level label rather than a guessed species. Taxonova made fewer confident mistakes when tested against seven existing tools on physical mock communities, biome-simulated mocks, and paired amplicon–shotgun datasets, Taxonova runs on ordinary laptops without specialised hardware and is freely available as both a Python package and a web application, lowering the barrier for researchers to obtain species-rank labels from their own amplicon data.

## Introduction

Amplicon sequencing of ribosomal RNA genes remains the most widely used approach for characterizing microbial communities, particularly for samples with low bacterial biomass or when cost constraints preclude shotgun metagenomics [1]. However, accurate taxonomic classification at species level represents a persistent bottleneck. This limitation stems from three interconnected challenges: the intrinsic information content of amplicon sequences, the structure of reference databases, and the classification algorithms applied to these data.

### The phylogenetic resolution problem

Standard 16S rRNA amplicons spanning one or two variable regions (V4: ~ 250 bp; V3-V4: ~ 450 bp) contain insufficient phylogenetic signal to distinguish closely related species [2]. While full-length 16S sequences (~1500 bp) and multi-gene ribosomal operons (~4900 bp) offer better resolution, short-read amplicons remain dominant due to established protocols and lower sequencing costs. The challenge is extracting maximum taxonomic information from these limited sequence windows. Long-read full-length 16S sequencing can resolve intragenomic copy-number variation and recover species-level differences invisible to short variable regions [3], and large-scale clinical evaluations of full-length 16S report approximately 91% species-level concordance against best-match references [4]. Even for short-read amplicons, much of the gap is reference-driven rather than intrinsic: ecosystem-specific high-resolution reference databases substantially improve genus- and species-level calls from V1-V3, V3-V4, V4 and V5-V7 reads [5]. These results frame species-level 16S classification as tractable but conditional on sequencing length, reference completeness, and the classifier, and motivate the classifier design and benchmarking strategy taken in this work.

### Reference database limitations

Taxonomic reference databases such as Greengenes2 [6] and SILVA [7] suffer from extreme class imbalance. Well-studied environments (such as the human gut) contribute thousands of reference sequences per species, while taxa from underexplored habitats may have only single representatives. This skewed distribution creates two problems for classification: models overfit to abundant taxa, and rare species are systematically misclassified or assigned to more common relatives [8]. Additionally, incomplete taxonomic annotation means many sequences lack species-level labels, forcing classifiers to handle partial information.

### Limitations of current classification approaches

Existing taxonomic classifiers fall into three categories, each with distinct limitations:

Similarity-based methods (BLASTn [9], VSEARCH [10]) align query sequences against reference databases and transfer taxonomy from the closest matches. These approaches work well when queries closely match database entries but degrade rapidly with increasing divergence. For short amplicons where many species share near-identical sequences, similarity searches often return ambiguous results or incorrect assignments.

Machine learning classifiers treat taxonomy assignment as a classification problem. The QIIME 2 naive Bayes classifier [2] (also implemented in Mothur [11] and RDP-Classifier [12]), IDTAXA [13], and Kraken 2 [14] each learn to map sequence features to taxonomic labels. These methods commit to a single assignment at the most specific rank (usually species or genus), accumulate confidence upward through the taxonomy, and truncate the reported lineage at the deepest rank whose confidence reaches a threshold. The depth of the reported lineage therefore depends on this threshold, and two distinct error modes, mis-classification (assigning the wrong taxon when the correct taxon is in the reference) and over-classification (assigning any taxon when the correct one is absent from the reference), must be measured separately; the distinction was made explicit by Edgar [15] and quantified separately in IDTAXA [13] and the QIIME 2 evaluation [2].

Phylogenetic placement methods place query sequences into a fixed reference phylogeny by maximum likelihood rather than predicting a single taxonomic label. pplacer [16] and the more recent EPA-ng [17] score each query against branches of a curated reference tree and report the most likely placement(s) with posterior support; the taxonomy returned is the lineage of the placed branch. TIPP [18] and its successor TIPP2 [19] build on this idea with marker-gene-based binning and statistical support that permits confident placement only at the rank the phylogeny supports, effectively an explicit form of abstention. Phylogenetic placement has two practical advantages relevant here: it provides a tree-aware notion of confidence (a query that lands on a deep branch is reported as such rather than forced to a leaf), and it tends to over-classify less on queries from taxa absent from the reference, because such queries naturally place on internal branches. Its costs are computational, placement is substantially slower than *k*-mer or alignment methods, the reference phylogeny must be curated and maintained, and the achievable resolution is fundamentally capped by the resolution of that tree.

We developed Taxonova, a deep learning classifier for ribosomal amplicons ranging from single variable regions to the full ribosomal operon. Taxonova computes a confidence at each taxonomic level and reports the lineage only down to the deepest level whose confidence meets its threshold, so that a sequence whose species cannot be resolved is reported at genus level rather than assigned a guessed species. To handle class imbalance in reference databases, we implemented a focal loss function [20] that dynamically down-weights well-classified (typically abundant) taxa during training.

## Results

The evaluation is organised in three tiers of increasing realism but decreasing controllability. The physical mocks, Mockrobiota [21], plus an in-house long-read mock for gut (gutmock) and vaginal (vaginalmock) communities, provide a defined composition that is independent of any reference database and serves as an external cross-check. The biome-simulated mocks (digestive system, lung, sputum, skin, soil, plants and a mixed-biome control) are built from Greengenes2 sequences sampled at biome-realistic genus abundances derived from MGnify [22] (see Materials and Methods). The paired amplicon–shotgun studies (Zuo *et al.* [23]; Mantri *et al.* [24]; Virtanen *et al.* [25]) provide a real-world test where shotgun metagenomics, free of PCR primer bias, serves as an independent reference taxonomy. Tier 1 establishes a noise-free upper bound, tier 2 systematically varies biome composition, and tier 3 tests transferability to the field.

### Species-level performance

Taxonova achieved consistently high performance across all evaluated amplicon lengths and biome contexts, with species-level F-measures spanning 0.76–1.00 across the 30 short-amplicon sequences (V4, V3–V4, V5–V7; median 0.94) and 0.82–0.99 across the 17 long-amplicon sequences (whole 16S, RRN; median 0.97) (Fig 1a, 1b; Tables 1, 2, S1 Fig). Across the three physical mocks (Mockrobiota-16S, gutmock, vaginalmock) and the seven biome-simulated communities, Taxonova’s species-level precision spanned 0.77–1.00 (median 0.97), and recall spanned 0.76–0.99 (median 0.95).

**Fig 1:**
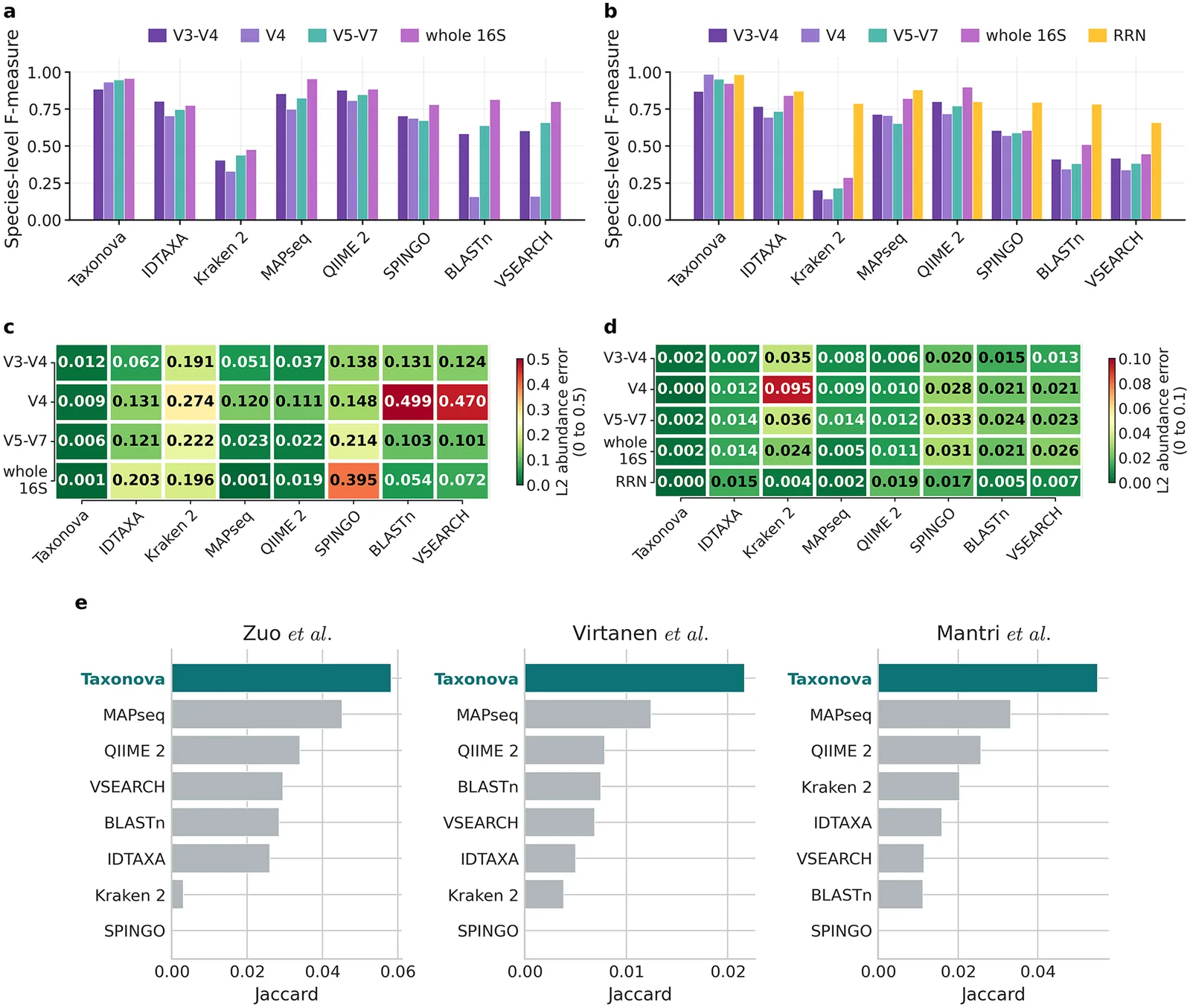
Species-level accuracy and real-world concordance across amplicon regions and classifiers. **(a)** Species-level F-measure (harmonic mean of precision and recall) on the three physical mock communities (Mockrobiota-16S, gutmock, vaginalmock), grouped by classifier and coloured by amplicon region. **(b)** Species-level F-measure on the seven biome-simulated short-amplicon mocks and the matched full-operon (RRN, ~ 4,900 bp) test sets. Taxonova maintains high performance across amplicon lengths, whereas similarity-based methods (BLASTn, VSEARCH) degrade sharply on short amplicons. **(c)** Relative-abundance estimation error (per-rank L2 norm) on the physical mocks and (d) on the biome-simulated mocks and RRN sets; lower is better, and Taxonova (leftmost column) shows the smallest residuals in every region. **(e)** Species-level Jaccard concordance between amplicon-derived and paired shotgun profiles for the three real-world studies (Zuo *et al*., Virtanen *et al*., Mantri *et al*.), with classifiers ranked within each study; Taxonova ranks highest in all three.

**Table 1:** Species-level classification performance across three mock communities.

Mock Community	Region	Metric	Taxonova	QIIME 2	IDTAXA	MAPseq	SPINGO	Kraken 2	BLASTn	VSEARCH
Mockrobiota	V4	Recall	0.904	0.606	0.489	0.574	0.362	0.117	0.309	0.319
		Precision	0.924	0.966	0.939	0.844	0.523	0.344	0.853	0.833
		F-score	0.914	0.745	0.643	0.683	0.428	0.175	0.454	0.461
	V3-V4	Recall	0.831	0.712	0.542	0.695	0.407	0.186	0.475	0.466
		Precision	0.845	0.944	0.955	0.901	0.495	0.379	0.875	0.887
		F-score	0.838	0.812	0.692	0.785	0.447	0.25	0.616	0.611
	V5-V7	Recall	0.962	0.603	0.538	0.577	0.321	0.115	0.513	0.603
		Precision	0.974	0.959	1	0.865	0.439	0.243	0.816	0.959
		F-score	0.968	0.74	0.7	0.692	0.371	0.156	0.63	0.74
	whole 16S	Recall	0.901	0.711	0.662	0.859	0.458	0.197	0.732	0.718
		Precision	0.908	0.91	1	0.924	0.464	0.231	0.981	1
		F-score	0.904	0.798	0.797	0.89	0.461	0.213	0.838	0.836
Gutmock	V4	Recall	0.992	0.933	0.933	0.925	0.958	0.683	0.008	0.008
		Precision	0.992	0.991	1	0.974	0.975	1	1	1
		F-score	0.992	0.961	0.965	0.949	0.966	0.812	0.016	0.016
	V3-V4	Recall	0.953	0.982	0.982	0.929	0.971	0.753	0.359	0.359
		Precision	0.964	0.988	1	0.958	0.976	1	0.813	0.847
		F-score	0.958	0.985	0.991	0.943	0.973	0.859	0.498	0.504
	V5-V7	Recall	0.957	0.906	0.906	0.855	0.978	0.804	0.362	0.348
		Precision	0.957	0.984	1	0.975	0.978	1	0.725	0.727
		F-score	0.957	0.943	0.951	0.911	0.978	0.891	0.483	0.471
	whole 16S	Recall	0.987	0.982	0.964	0.987	0.987	0.883	0.525	0.475
		Precision	0.987	1	1	1	0.987	0.995	0.893	0.891
		F-score	0.987	0.991	0.982	0.993	0.987	0.936	0.661	0.62
Vaginalmock	V4	Recall	0.889	0.556	0.333	0.444	0.556	0	0	0
		Precision	0.889	1	1	1	0.833	0	0	0
		F-score	0.889	0.715	0.5	0.615	0.667	0	0	0
	V3-V4	Recall	0.857	0.714	0.571	0.714	0.643	0.071	0.5	0.571
		Precision	0.857	1	1	1	0.75	0.2	0.875	0.889
		F-score	0.857	0.833	0.727	0.833	0.692	0.105	0.636	0.695
	V5-V7	Recall	0.917	0.75	0.417	0.833	0.583	0.167	0.667	0.667
		Precision	0.917	1	1	0.909	0.778	0.667	1	0.889
		F-score	0.917	0.857	0.589	0.869	0.667	0.267	0.8	0.762
	whole 16S	Recall	0.979	0.812	0.375	0.979	0.875	0.229	0.896	0.896
		Precision	0.979	0.929	1	0.979	0.913	0.355	1	1
		F-score	0.979	0.867	0.545	0.979	0.894	0.278	0.945	0.945

**Table 2:** Species-level classification performance across seven simulated biomes.

Biome	Region	Metric	Taxonova	QIIME 2	IDTAXA	MAPseq	SPINGO	Kraken 2	BLASTn	VSEARCH
Digestive System	V4	Recall	0.984	0.646	0.606	0.772	0.457	0.11	0.291	0.283
		Precision	0.992	0.953	0.987	0.916	0.563	0.264	0.787	0.783
		F-score	0.988	0.77	0.751	0.838	0.504	0.155	0.425	0.416
	V3-V4	Recall	0.931	0.759	0.731	0.807	0.49	0.2	0.393	0.414
		Precision	0.938	0.932	0.972	0.9	0.559	0.319	0.781	0.75
		F-score	0.934	0.837	0.834	0.851	0.522	0.246	0.523	0.534
	V5-V7	Recall	0.932	0.78	0.746	0.644	0.542	0.237	0.288	0.305
		Precision	0.932	0.958	0.957	0.809	0.593	0.333	0.548	0.529
		F-score	0.932	0.86	0.838	0.717	0.566	0.277	0.378	0.387
	whole 16S	Recall	0.955	0.909	0.818	0.939	0.591	0.348	0.303	0.273
		Precision	0.969	1	1	0.954	0.591	0.39	0.769	0.75
		F-score	0.962	0.952	0.9	0.946	0.591	0.368	0.435	0.4
	RRN	Recall	0.974	0.744	0.824	0.91	0.79	0.786	0.746	0.55
		Precision	0.998	0.947	1	0.936	0.792	0.833	0.921	0.948
		F-score	0.986	0.833	0.904	0.923	0.791	0.809	0.824	0.696
Lung	V4	Recall	0.985	0.583	0.523	0.629	0.523	0.083	0.182	0.182
		Precision	0.992	0.975	1	0.902	0.69	0.239	0.571	0.615
		F-score	0.988	0.73	0.687	0.741	0.595	0.123	0.276	0.281
	V3-V4	Recall	0.864	0.636	0.617	0.649	0.578	0.162	0.273	0.286
		Precision	0.869	0.951	0.979	0.935	0.685	0.329	0.646	0.677
		F-score	0.866	0.762	0.757	0.766	0.627	0.217	0.384	0.402
	V5-V7	Recall	0.953	0.682	0.624	0.565	0.635	0.212	0.318	0.318
		Precision	0.953	0.951	0.964	0.842	0.74	0.36	0.6	0.562
		F-score	0.953	0.794	0.758	0.676	0.683	0.267	0.416	0.406
	whole 16S	Recall	0.88	0.837	0.707	0.87	0.717	0.348	0.326	0.239
		Precision	0.91	1	1	0.92	0.725	0.438	0.732	0.733
		F-score	0.895	0.911	0.828	0.894	0.721	0.388	0.451	0.36
	RRN	Recall	0.978	0.788	0.867	0.877	0.853	0.82	0.711	0.515
		Precision	0.992	0.963	1	0.891	0.854	0.908	0.926	0.944
		F-score	0.985	0.867	0.929	0.884	0.853	0.862	0.804	0.666
Mixed	V4	Recall	0.993	0.652	0.638	0.73	0.546	0.142	0.348	0.355
		Precision	1	0.939	1	0.88	0.636	0.27	0.803	0.806
		F-score	0.996	0.77	0.779	0.798	0.588	0.186	0.486	0.493
	V3-V4	Recall	0.93	0.764	0.745	0.758	0.573	0.191	0.414	0.427
		Precision	0.936	0.952	0.983	0.869	0.629	0.288	0.812	0.798
		F-score	0.933	0.848	0.848	0.81	0.6	0.23	0.548	0.556
	V5-V7	Recall	0.968	0.742	0.726	0.629	0.532	0.21	0.387	0.403
		Precision	0.968	0.958	0.957	0.78	0.623	0.31	0.585	0.625
		F-score	0.968	0.836	0.826	0.696	0.574	0.25	0.466	0.49
	whole 16S	Recall	0.955	0.894	0.788	0.909	0.667	0.318	0.424	0.348
		Precision	0.984	1	1	0.923	0.677	0.35	0.875	0.821
		F-score	0.969	0.944	0.881	0.916	0.672	0.333	0.571	0.489
	RRN	Recall	0.97	0.812	0.882	0.92	0.846	0.834	0.666	0.486
		Precision	0.994	0.964	1	0.939	0.848	0.882	0.915	0.931
		F-score	0.982	0.881	0.937	0.929	0.847	0.857	0.771	0.639
Plants	V4	Recall	0.947	0.473	0.458	0.443	0.412	0.061	0.191	0.176
		Precision	0.947	0.984	1	0.763	0.628	0.205	0.641	0.548
		F-score	0.947	0.639	0.628	0.561	0.498	0.094	0.294	0.266
	V3-V4	Recall	0.812	0.674	0.597	0.438	0.542	0.132	0.201	0.215
		Precision	0.824	0.96	0.966	0.778	0.65	0.224	0.592	0.544
		F-score	0.818	0.792	0.738	0.56	0.591	0.166	0.3	0.308
	V5-V7	Recall	0.986	0.587	0.517	0.51	0.455	0.091	0.294	0.294
		Precision	0.986	0.977	0.987	0.768	0.596	0.178	0.7	0.677
		F-score	0.986	0.733	0.679	0.613	0.516	0.12	0.414	0.41
	whole 16S	Recall	0.957	0.675	0.692	0.516	0.159	0.044	0.833	0.792
		Precision	0.974	0.889	1	0.616	0.16	0.049	0.967	0.997
		F-score	0.965	0.767	0.818	0.562	0.159	0.046	0.895	0.883
	RRN	Recall	0.943	0.225	0.339	0.64	0.524	0.349	0.767	0.751
		Precision	0.982	0.833	1	0.693	0.525	0.434	0.943	0.986
		F-score	0.962	0.354	0.506	0.665	0.524	0.387	0.846	0.853
Skin	V4	Recall	0.993	0.559	0.493	0.551	0.574	0.132	0.125	0.132
		Precision	1	0.974	1	0.893	0.757	0.409	0.708	0.667
		F-score	0.996	0.71	0.66	0.682	0.653	0.2	0.212	0.22
	V3-V4	Recall	0.844	0.619	0.556	0.506	0.613	0.169	0.188	0.194
		Precision	0.849	0.952	0.978	0.9	0.731	0.346	0.698	0.705
		F-score	0.846	0.75	0.709	0.648	0.667	0.227	0.296	0.304
	V5-V7	Recall	0.915	0.617	0.553	0.521	0.543	0.181	0.191	0.202
		Precision	0.915	0.879	0.929	0.79	0.708	0.362	0.486	0.475
		F-score	0.915	0.725	0.693	0.628	0.615	0.241	0.274	0.283
	whole 16S	Recall	0.876	0.856	0.711	0.814	0.711	0.34	0.206	0.144
		Precision	0.895	1	1	0.919	0.719	0.452	0.645	0.667
		F-score	0.885	0.922	0.831	0.863	0.715	0.388	0.312	0.237
	RRN	Recall	0.984	0.821	0.888	0.922	0.851	0.884	0.649	0.402
		Precision	0.998	0.969	1	0.939	0.851	0.926	0.915	0.935
		F-score	0.991	0.889	0.941	0.93	0.851	0.905	0.759	0.562
Soil	V4	Recall	0.992	0.46	0.419	0.371	0.5	0.065	0.153	0.137
		Precision	0.992	0.95	1	0.742	0.721	0.242	0.679	0.607
		F-score	0.992	0.62	0.591	0.495	0.59	0.102	0.25	0.224
	V3-V4	Recall	0.756	0.635	0.538	0.353	0.583	0.109	0.154	0.16
		Precision	0.766	0.98	0.988	0.775	0.7	0.221	0.649	0.568
		F-score	0.761	0.771	0.697	0.485	0.636	0.146	0.249	0.25
	V5-V7	Recall	0.972	0.486	0.403	0.403	0.472	0.111	0.167	0.139
		Precision	0.972	0.972	0.967	0.784	0.68	0.229	0.545	0.435
		F-score	0.972	0.648	0.569	0.532	0.557	0.15	0.256	0.211
	whole 16S	Recall	0.813	0.827	0.613	0.6	0.693	0.227	0.213	0.147
		Precision	0.836	1	1	0.726	0.712	0.288	0.727	0.733
		F-score	0.824	0.905	0.76	0.657	0.702	0.254	0.329	0.245
	RRN	Recall	0.976	0.792	0.87	0.888	0.838	0.798	0.548	0.342
		Precision	0.994	0.975	1	0.901	0.838	0.873	0.923	0.891
		F-score	0.985	0.874	0.93	0.894	0.838	0.834	0.688	0.494
Sputum	V4	Recall	0.986	0.674	0.617	0.766	0.511	0.099	0.333	0.333
		Precision	0.993	0.95	0.989	0.893	0.626	0.215	0.77	0.758
		F-score	0.989	0.789	0.76	0.825	0.563	0.136	0.465	0.463
	V3-V4	Recall	0.92	0.759	0.673	0.833	0.562	0.154	0.457	0.463
		Precision	0.925	0.939	0.973	0.925	0.632	0.25	0.796	0.75
		F-score	0.922	0.839	0.796	0.877	0.595	0.191	0.581	0.573
	V5-V7	Recall	0.941	0.691	0.647	0.632	0.574	0.162	0.382	0.412
		Precision	0.941	0.959	0.957	0.782	0.65	0.256	0.591	0.622
		F-score	0.941	0.803	0.772	0.699	0.61	0.198	0.464	0.496
	whole 16S	Recall	0.946	0.824	0.77	0.905	0.676	0.216	0.446	0.378
		Precision	0.972	0.968	1	0.918	0.685	0.25	0.825	0.824
		F-score	0.959	0.89	0.87	0.911	0.68	0.232	0.579	0.518
	RRN	Recall	0.984	0.824	0.893	0.929	0.864	0.83	0.696	0.549
		Precision	0.996	0.974	1	0.948	0.87	0.876	0.917	0.951
		F-score	0.99	0.893	0.943	0.938	0.867	0.852	0.791	0.696

Recall = fraction of true species correctly detected.

Three physical mocks (Mockrobiota-16S [21], gutmock and vaginalmock, the latter two are full-length 16S mocks of the canonical paediatric-gut and vaginal communities, respectively) provide a reference-database-independent cross-check. On Mockrobiota-16S, Taxonova reached species-F = 0.914 (V4, ~ 250 bp), 0.838 (V3–V4, ~ 450 bp), 0.968 (V5–V7, ~ 395 bp) and 0.904 (whole 16S, ~ 1,500 bp); on gutmock, 0.992 / 0.958 / 0.957 / 0.987 at V4 / V3–V4 / V5–V7 / whole 16S; on vaginalmock, 0.889 / 0.857 / 0.917 / 0.979 at V4 / V3–V4 / V5–V7 / whole 16S. Across the physical mocks, Taxonova’s lowest species-F was 0.838 (Mockrobiota-16S at V3–V4) (Fig 1a; Tables 1, S2 Table).

Seven biome-simulated short-amplicon mocks (digestive system, lung, sputum, skin, soil, plants and a mixed-biome control) span a wider biological complexity range. Across the 28 short-amplicon sequences (7 biomes × {V4, V3–V4, V5–V7, whole 16S}), Taxonova species-F ranged from 0.761 (biome_soil at V3–V4 — the deepest-bottleneck sequence) to 0.996 (biome_mixed and biome_skin at V4), with per-region medians of 0.989 (V4), 0.866 (V3–V4), 0.953 (V5–V7) and 0.959 (whole 16S). For the seven matched full-operon (RRN, ~ 4,900 bp) test sets drawn from GROND, Taxonova species-F ranged 0.962 (gtdb_plants) to 0.991 (gtdb_skin), pushing performance close to the panel ceiling regardless of biome (Fig 1b; Tables 2, S1 Table).

### Plant-microbiome region (V5–V7,  ~~ 395 bp)

Plant microbiome studies frequently use the V5–V7 primer pair 799F/1193R because the 799F primer mismatches chloroplast 16S and the 1193R primer minimises mitochondrial co-amplification, dramatically reducing host contamination [26,27]. We trained a dedicated V5–V7 Taxonova model and benchmarked it on the full mock panel. Taxonova produced the lowest species-level false-positive rate of any tested method on Mockrobiota-16S and the seven biome-simulated mocks at V5–V7 (0.8–2.3 × 10^−4^, compared with 3.8–41.2 × 10^−4^ for the other seven classifiers; S10 Table), and the highest species-level F-measure of the eight classifiers on nine of the ten V5–V7 mock communities (Tables 1, 2). The exception was gutmock, where SPINGO had a higher species-level F-measure than Taxonova (0.978 compared to 0.957) and three classifiers had a lower species-level false-positive rate than Taxonova’s 1.5 × 10^−4^ (naive Bayes 0.0 × 10^−4^, Kraken 2 and IDTAXA 0.4 × 10^−4^; S3 Table).

### Paired amplicon-shotgun performance

Mock communities provide controlled evaluation but may not capture real-world complexity. We therefore compared amplicon-derived taxonomic profiles to paired shotgun metagenomic data, which avoids PCR amplification bias and provides an independent species-rank reference, across three studies spanning gut, soil and vaginal microbiomes, and reported two complementary headline metrics per study: species-rank Jaccard (presence/absence overlap) and species-rank Bray–Curtis (abundance-weighted compositional distance). Per-study species-level community compositions are shown in S1 and S2 Figs, the ranked species-level Jaccard across the three studies is summarised in Fig 1e, and the per-sample values are in S9 Table.

### High-complexity gut microbiome

On 42 paired paediatric ulcerative-colitis samples from Zuo *et al.* [23], Taxonova led species-rank Jaccard (0.058 vs. 0.045 for the next-best classifier, mapseq; paired mean difference 0.013, 95% CI 0.011 to 0.015) and significantly outperformed mapseq on Bray–Curtis (0.579 vs. 0.671, ΔBC = −0.092, 95% CI −0.115 to −0.069, paired Wilcoxon *p* < 0.001, 39 / 42 paired samples favouring Taxonova). The recovered species are the canonical pediatric-gut taxa of this disease context — *Faecalibacterium prausnitzii*, *Phocaeicola vulgatus*, *Bacteroides fragilis*, *Bifidobacterium adolescentis*, *Agathobacter rectalis* and *Ruminococcus bromii* — including several where Taxonova transparently reports an amplicon-indistinguishable bracket (e.g., `[F. duncaniae | F. prausnitzii]`; see Materials and Methods).

### Soil microbiome

On 7 paired Cambisol/Podzol/Stagnosol samples from Mantri *et al.* [24], Taxonova again led Jaccard (0.055 vs. 0.033 for mapseq; paired mean difference 0.022, 95% CI 0.018 to 0.026) and significantly outperformed mapseq on Bray–Curtis (0.856 vs. 0.897, ΔBC = −0.041, 95% CI −0.045 to −0.037, *p* = 0.022, all 7 / 7 paired samples favouring Taxonova). The recovered taxa are Cambisol/Podzol-appropriate residents (*Trebonia kvetii*, *Bradyrhizobium ottawaense / neotropicale*, *Conexibacter woesei*, *Edaphobacter aggregans*, *Paraburkholderia xenovorans*).

### Low-complexity vaginal microbiome

On 21 paired vaginal-microbiome samples from Virtanen *et al.* [25], Taxonova led Jaccard by a wide margin (0.022 vs. mapseq 0.012; paired mean difference 0.009, 95% CI 0.005 to 0.013) and by 0.015 over VSEARCH (0.007), recovering the canonical *Lactobacillus*-dominant and BV-associated taxa (*L. crispatus* — reported as the bracket `[L. crispatus | Ureaplasma parvum]` where the V3–V4 region cannot uniquely discriminate the two — *L. iners*, *L. jensenii*, *Bifidobacterium vaginale* (formerly *Gardnerella vaginalis*), *Fannyhessea vaginae*, *Megasphaera lornae*). On Bray–Curtis, the four top classifiers — VSEARCH, BLAST, Taxonova and mapseq — cluster in a statistically indistinguishable ~0.03-wide band (pairwise Wilcoxon *p* ≥ 0.21). The per-classifier per-sample BC standard deviation (≈ 0.19) is roughly six times the band width, so on this study no classifier wins Bray–Curtis. By contrast, the Jaccard signal is clean, which illustrates that in the low-overlap regime where amplicon-vs-shotgun Jaccard is intrinsically ≤ 0.10 across the panel (a property of the underlying biology, not of any classifier), Bray–Curtis can saturate into a noisy null while Jaccard remains discriminating. Reporting both metrics is therefore necessary, and the two together capture more than either alone. The full per-study ranked Bray–Curtis comparison across all classifiers is shown in S3 Fig.

The real-world comparison also makes an important caveat tangible: PCR primer bias physically excludes certain taxa from the sequencing library before classification, so no classifier (regardless of accuracy) can detect species that were never amplified. The observed species-rank Jaccard indices of 0.02–0.06 reflect that fundamental constraint of amplicon sequencing rather than classifier performance alone and explain the absolute scale on which the per-classifier differences play out.

### Error modes: mis-classification, over-classification, and abstention

Two distinct error modes affect microbiome classifiers differently: assigning the wrong taxon when the correct one is present in the reference (mis-classification, MC), and assigning any taxon when the correct one is absent from the reference (over-classification, OC) [2,13]. We here quantified both error types in two complementary settings; abundance-estimation accuracy (L1 and L2 norms) is a separate outcome and is reported in the next section (“Abundance estimation accuracy”).

**Mis-classification on mock communities.** On the mock communities, where every true taxon is in the reference, MC is the only failure mode that can occur. Taxonova had the lowest species-level false-positive rate of the eight classifiers in 38 of the 39 test sets for which S10 Table gives all eight classifiers (the exception was biome_soil at whole 16S, where naive Bayes was lower; on gutmock at V5–V7, which S10 Table gives for Taxonova only, three classifiers were lower, see “Plant-microbiome region” and S3 Table), and the median difference between another classifier’s false-positive rate and Taxonova’s was 16.2 × 10^−4^ (per-classifier medians 7.7 × 10^−4^ for IDTAXA to 40.4 × 10^−4^ for SPINGO; S1, S10 Tables). On the biome_digestive V4 cell, for example, Taxonova reached species-level specificity of 0.99990 (false-positive rate 1.0 × 10^−4^) versus 0.99881 for MAPseq, 0.99823 for naive Bayes and 0.99804 for IDTAXA, with the corresponding false-positive rates 10.9 × 10^−4^, 16.7 × 10^−4^ and 18.6 × 10^−4^ higher than Taxonova’s. Species-level specificity (TN / (TN + FP), where the negative universe is the per-region reference vocabulary) is correspondingly the highest of any tested classifier in the same 38 test sets (S1, S10 Tables).

**Over-classification in leave-out analyses.** Over-classification cannot occur on mock communities, because every species in them is in the reference. We therefore built, for each amplicon region, a leave-one-clade-out reference by removing 200 species, 100 genera, 50 families, 25 orders and 10 classes, and retrained or re-indexed all eight classifiers on it (Taxonova, IDTAXA, QIIME 2, BLASTn, VSEARCH, SPINGO, Kraken 2 and MAPseq; Materials and methods). For each rank, we measured the MC rate on control sequences whose taxa remain in the reference, the OC rate on sequences from the removed taxa, and the under-classification (UC) rate, the fraction of control sequences that receive no assignment at that rank. Because confidence values are not comparable between classifiers or between regions, MC and OC were compared at the same UC rate rather than at the same confidence threshold, following Murali *et al.* [13] (Figs 2; S4).

**Fig 2:**
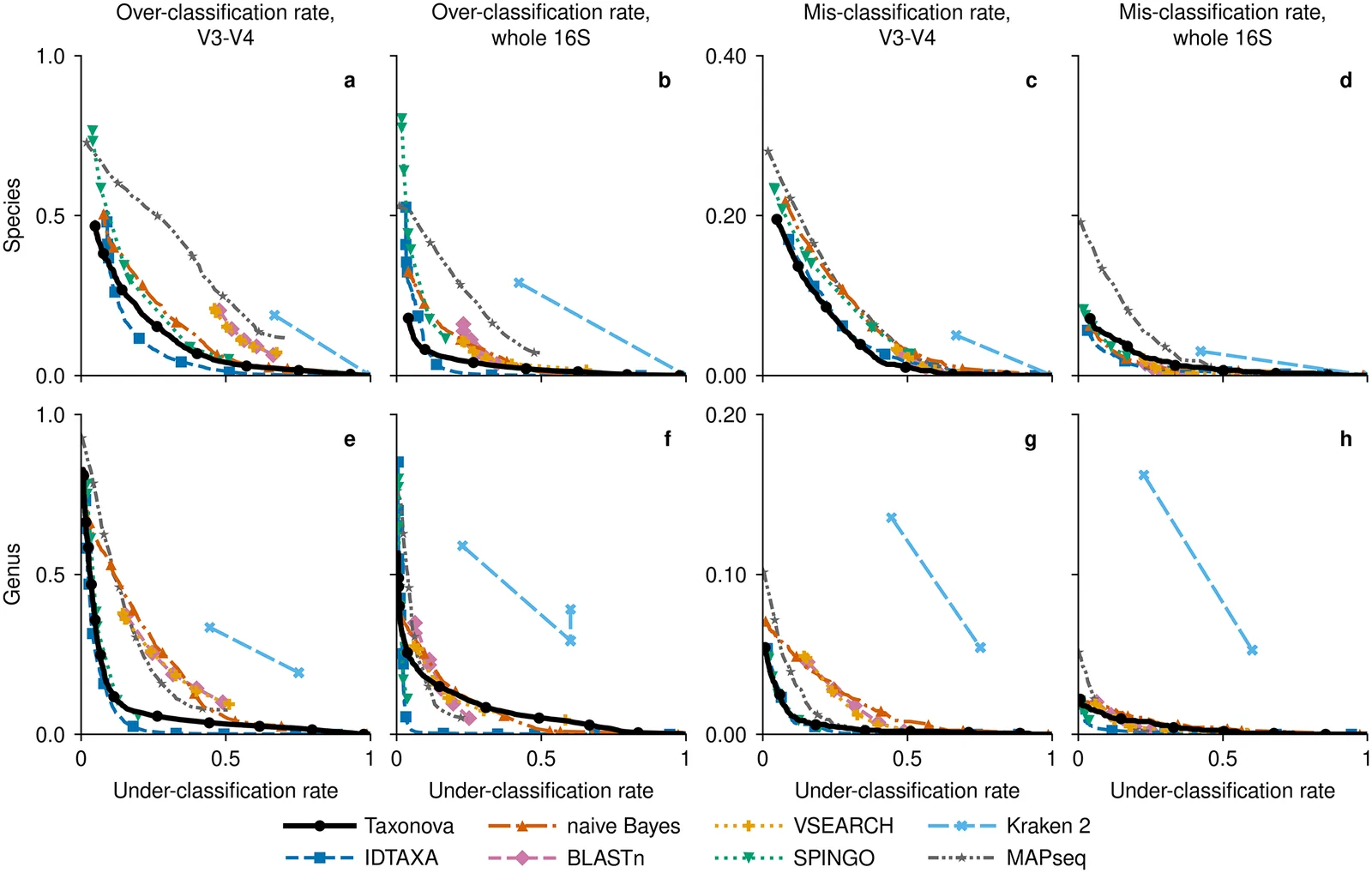
Leave-one-clade-out error curves at V3–V4 and whole 16S at species and genus rank. Over-classification rate (a, b, e, f: the fraction of novel sequences that received a confident assignment at the rank shown) and mis-classification rate (c, d, g, h: the fraction of control sequences that received a confident but wrong assignment at that rank), plotted against the under-classification rate at species (a–d) and genus rank **(e–h)**, for all eight classifiers in the leave-out analysis with 200 species removed. The confidence threshold is the parameter along each curve and is not plotted. The scale of the mis-classification axis differs between the two ranks. In the legend, naive Bayes is the QIIME 2 classifier. The other three regions, and family, order and class rank, are shown in S4 and S8–S11 Figs.

At a UC rate of 0.50, Taxonova’s species-level OC and MC rates were 0.043 and 0.056 at V4, 0.041 and 0.010 at V3–V4, 0.021 and 0.006 at V5–V7, 0.017 and 0.006 at whole 16S, and 0.022 and 0.002 at RRN, so its species-level OC was below 5% in every region. Of the eight classifiers, Taxonova had the lowest species-level MC at V3–V4, V5–V7 and RRN and the lowest OC at RRN, where its OC of 0.022 was 0.123 lower than that of the next-best classifier, VSEARCH (0.146), and 0.185 lower than that of IDTAXA (0.207; differences calculated from the unrounded rates). In the four 16S regions, IDTAXA had lower species-level OC than Taxonova (0.039, 0.013, 0.011 and 0.000, compared to 0.043, 0.041, 0.021 and 0.017) but higher MC at V4 (0.065 compared to 0.056), V3–V4 (0.017 compared to 0.010) and V5–V7 (0.014 compared to 0.006), so neither classifier had lower rates of both errors in these three regions; at whole 16S, IDTAXA had lower rates of both (MC 0.005 compared to 0.006). S5 Table gives the results for every region, classifier and rank; Fig 2 shows the species- and genus-level curves at V3–V4 and whole 16S; S4 Fig shows the species-level curves for all five regions, and S8–S11 Figs show the curves at genus, family, order and class rank.

**Leave-out analysis with 2,000 species removed.** To test whether species-level OC stays below 5% when more of the reference is removed, we repeated the analysis after removing 2,000 species instead of 200, with three independent random selections of species (S13 Table). At a UC rate of 0.50, Taxonova’s species-level OC was 0.087 at V4, and across the three selections averaged 0.067 (SD 0.019) at V3–V4, 0.055 (SD 0.008) at V5–V7, 0.031 (SD 0.012) at whole 16S and 0.042 (SD 0.015) at RRN. It therefore exceeded 5% at V4, V3–V4 and V5–V7. The differences between selections were similar in size to the differences between regions. At a UC rate of 0.65, species-level OC was below 5% in every region and every selection (highest value 0.045). At V4 only 1,817 species could be removed, so the three selections at V4 were identical (Materials and methods). Removing 2,000 species also removed 17–33% of the reference sequences, so the 200-species and 2,000-species analyses differ in reference size as well as in the number of removed species. At RRN, Taxonova’s species-level OC at a UC rate of 0.50 remained the lowest of the eight classifiers, 0.077 lower than that of the next-best classifier, IDTAXA (0.051 compared to 0.127; difference calculated from the unrounded rates in S13 Table (a)). S13 Table (b) gives, for each classifier and region, the lowest UC rate at which species-level OC falls below 5%. OC, and not MC, is the error that applies to species absent from the reference. In both leave-out analyses, IDTAXA had lower species-level OC than Taxonova in the four 16S regions at a UC rate of 0.50 (Figs 2a, 2b; S4; S13 Table (a)), thus the false-positive rates measured on the mock communities, in which every species is in the reference, describe only communities whose species are represented in the reference.

**Failure-mode profile of incorrect calls.** Error analysis on the mock panels confirms the same pattern at the level of individual predictions. When Taxonova made an incorrect species call, it predominantly underclassified (correct genus, no species call) rather than mis-classified (wrong species). This is the intended behaviour of the confidence-aware fallback (see Materials and Methods): when species-level evidence is ambiguous, Taxonova reports a genus-level label rather than commit to one of several indistinguishable candidates. Methods without confidence thresholds frequently produced confident but incorrect species assignments.

**Robustness to reference-set label noise.** Because public taxonomic references carry non-trivial annotation error [40], we retrained Taxonova on V4 references corrupted with two forms of synthetic label noise — species mis-labelling within a genus, and genus misplacement within a family — at injected rates of 1–20%, and re-evaluated each retrain on the held-out clean mock panel (means across the seven V4 biome mocks). Degradation was gradual: from a clean-reference mean species-level F-measure of 0.97, species F fell to 0.93 (species-within-genus) and 0.91 (genus-within-family) at 10% injected noise — near the ~ 10% error empirically reported for public references — and to 0.88 and 0.83 at 20%. The error did not cascade upward: genus-level F-measure stayed ≥ 0.91 at every rate, and under species-level mis-labelling it was essentially unchanged (0.99; S11 Table, S7 Fig).

### Generalisation to references other than Greengenes2

All results above use Greengenes2 (V4 to whole 16S) or GROND (RRN) as the reference. To test Taxonova with other references, we ran the TAXXI benchmark of Edgar [55]. Its training and test sets are drawn from NCBI, RDP and Warcup references of 1,000–4,000 sequences, two to three orders of magnitude smaller than the references used above, and include a dataset of the fungal internal transcribed spacer (ITS).

We scored all 80 training and test runs with the metric definitions distributed with the benchmark (Materials and methods). At species rank, 19 combinations of dataset and identity level had at least ten test sequences whose species was present in the training set, the minimum the benchmark applies. Comparing Taxonova with each of the seven classifiers evaluated in the benchmark in these 19 combinations gave 133 comparisons (S15 Table). At the same under-classification rate, Taxonova achieved higher species accuracy than the other classifier in 43 comparisons and lower mis-classification in 54. With Taxonova at the confidence thresholds used throughout this study (0.5 for domain to genus and 0.7 for species) and the other classifiers at the settings Edgar reports (for RDP-Classifier, 50% and 80% bootstrap support), Taxonova had the highest species accuracy of the eight classifiers in 12 combinations and the joint highest in 3, including all seven combinations at 99% identity (S15 Table). These seven have 177–6,097 test sequences with a known species each; the other twelve have 10–122, and in many of them every classifier had a species accuracy of zero.

The largest combination is the fungal ITS dataset at 99% identity, with 6,097 test sequences with a known species. Here, Taxonova had higher species accuracy than every other classifier at the same under-classification rate (71.1%, compared to 70.3% for RDP-Classifier at 80%, the next highest) and lower mis-classification (4.1% compared to 4.8%), with similar under-classification (10.2% and 10.0%).

We also calculated over-classification on this benchmark (S15 Table) and compared Taxonova’s over-classification and mis-classification rates, as a function of its under-classification rate, with those of the classifiers evaluated in the benchmark (S12 Fig). At the same under-classification rate, Taxonova’s over-classification was lower than that of the other classifier in 64 of the 133 comparisons, equal in 14 and higher in 55; RDP-Classifier at 80% had lower over-classification rates than Taxonova in 18 of the 19 combinations.

This comparison also depends on how often each classifier abstains. On the `ten_16s` dataset at 99% identity, where Taxonova ranked first at these thresholds, it left 55.7% of the test sequences with a known species unassigned, compared to 5.8% for RDP-Classifier at 80% (S15 Table). Edgar [55] reports that no classifier in the benchmark exceeded 20% species accuracy on V4 or 30% on full-length 16S sequences, so low absolute accuracies are expected in this benchmark. Thus, Taxonova’s ranking relative to other classifiers is not specific to Greengenes2, but on these references, as on Greengenes2, it depends on whether classifiers are compared at fixed thresholds or at the same under-classification rate.

### Abundance estimation accuracy

Beyond classification accuracy — which addresses *which* taxa are called and is reported in the preceding section (“Error modes”) — many microbiome studies aim for accurate quantification of *how much* of each taxon is present. We measured abundance estimation accuracy using the per-rank L1 norm (sum of absolute differences between observed and expected relative abundances) and the matched L2 norm (root-sum-of-squares of the same residuals); the two are reported jointly so that broad and concentrated abundance errors are both visible (Figs 1c, 1d; S5, S6 Table).

Taxonova reduced L1-norm abundance error across all amplicon types, by a median of 0.52 to 1.50 depending on amplicon region, on a scale of 0–2. On V4 amplicons averaged over the seven biome mocks, mean species-level L1 was 0.035 for Taxonova versus 0.79–2.00 for the seven competing classifiers (per-classifier differences: 0.76 MAPseq, 0.81 naive Bayes, 0.91 IDTAXA, 1.50 BLASTn, 1.51 VSEARCH, 1.77 Kraken 2, 1.97 SPINGO; median 1.50). Per-region median differences in mean species-level L1 between the other classifiers and Taxonova were 1.50 (V4), 1.31 (V5–V7), 1.12 (V3–V4), 1.04 (whole 16S) and 0.52 (RRN). L2-norm errors were also lowest for Taxonova — Taxonova mean L2 = 2.6 × 10^−4^ versus 4.2 × 10^−3^ (MAPseq) to 9.5 × 10^−2^ (Kraken 2), confirming that Taxonova’s gains apply uniformly across the abundance distribution rather than being driven by a small number of large-error taxa (S6 Table). This improvement in abundance estimation has practical implications: differential abundance analyses depend on accurate quantification, and systematic abundance biases can lead to spurious biomarker discoveries.

Part of the abundance improvement may reflect the confidence-aware fallback: where species-level confidence is low, sequences are reported at genus level rather than assigned to a species, which avoids inflating the abundances of closely related species.

### Computational efficiency

Practical adoption requires reasonable computational resources. We measured runtime and memory usage across dataset sizes (Figs 3a, 3b).

**Fig 3:**
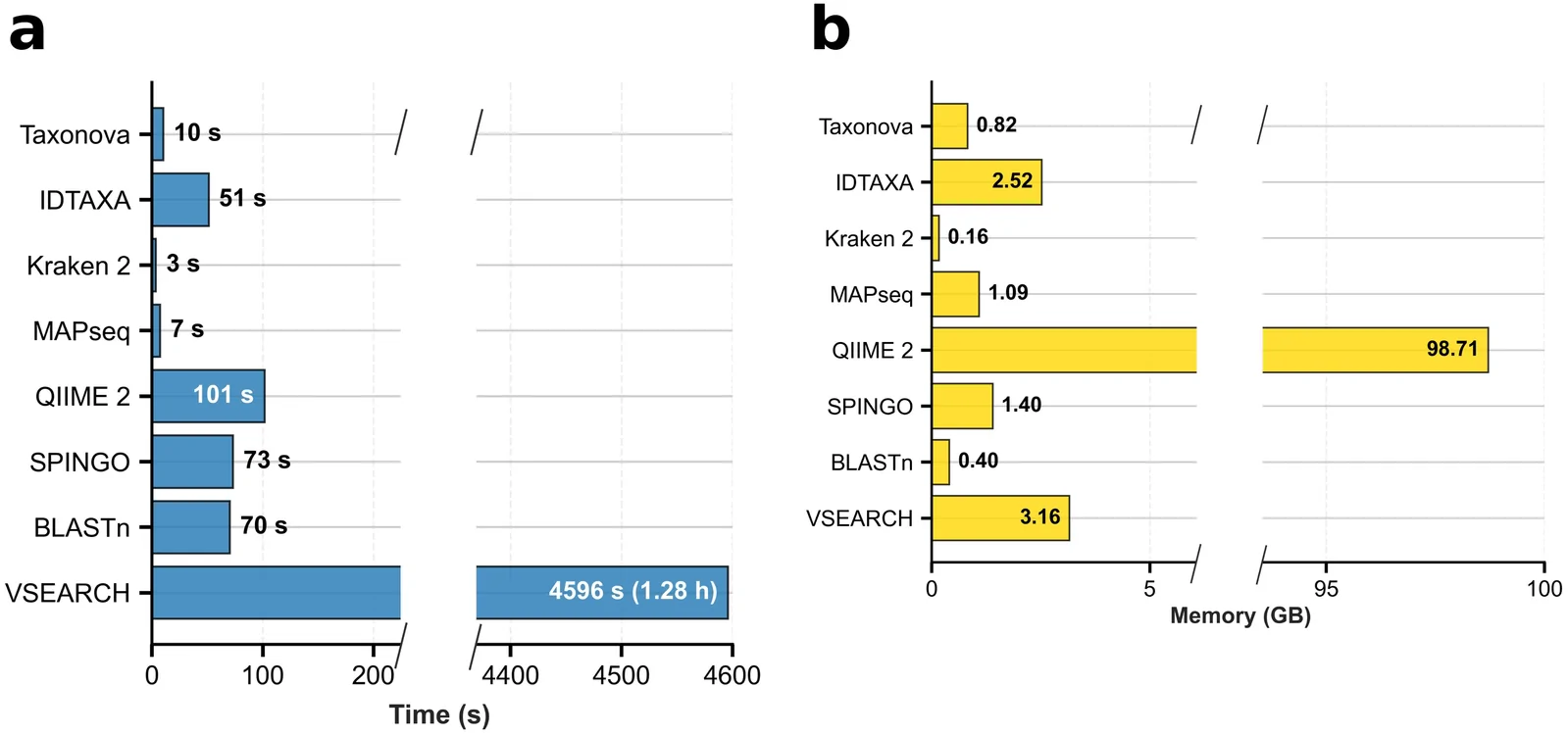
Computational efficiency. **(a)** Runtime to classify 1,000 sequences (seconds); the axis is broken to accommodate VSEARCH (4,596 s ≈ 1.28 **h)**. **(b)** Peak memory consumption (GB); the axis is broken to accommodate QIIME 2 (98.71 GB). Taxonova combines near-fastest runtime (10 s) with the lowest peak memory (0.82 GB) among the accurate classifiers, and its memory footprint is constant in dataset size because sequences are processed individually rather than loaded simultaneously.

**Runtime.** Taxonova processed 1000 sequences in 10.4 seconds (96 sequences/second), comparable to Kraken 2 (3.7 seconds) and substantially faster than QIIME 2’s naive Bayes classifier (98.7 seconds). The intermediate speed reflects Taxonova’s neural network inference, which is slower than *k*-mer lookup (Kraken 2) but faster than the naive Bayes approach, whose runtime is dominated by *k*-mer counting and the probability calculation over all lineages.

**Memory.** Taxonova required 0.82 GB peak memory regardless of dataset size, as sequences are processed individually rather than loaded into memory simultaneously. This contrasts with QIIME 2, which required 98.7 GB for the same analysis, limiting its use on standard computers.

**Hardware requirements.** Taxonova runs on CPU by default and does not require GPU hardware. Pre-trained models for all evaluated amplicon regions are provided, so users need not perform model training. For users who wish to train custom models (e.g., for novel amplicon regions or different reference databases), GPU acceleration reduces training time but is not strictly required.

### Comparison with existing methods

The seven compared methods represent distinct algorithmic approaches, and understanding their relative strengths and limitations contextualizes Taxonova’s contributions (Tables 1, 2).

The exact command-line invocations, version numbers and parameter values for every classifier are tabulated in S7 Table.

Similarity-based methods (BLASTn, VSEARCH) showed large performance variation across amplicon lengths. On RRN full-operon inputs (~4,900 bp, 7 gtdb biomes), BLASTn and VSEARCH reached species-F medians of 0.79 and 0.67 (max 0.85 each). For short V4 amplicons (~250 bp), the same methods dropped to median species-F of 0.36 and 0.35 (range 0.21–0.49 across 8 mocks), as limited sequence length increases the probability of spurious matches to incorrect taxa; the corresponding L1-norm abundance errors averaged 1.5 (compared to 0.035 for Taxonova).

*K*-mer matching methods (Kraken 2, MAPseq, SPINGO) varied widely. Kraken 2, originally designed for shotgun metagenomics, struggled severely with short amplicons (V4 species-F median 0.15) but recovered substantially for full operons (RRN median 0.85). MAPseq had species-F medians of 0.71 / 0.78 / 0.68 / 0.89 / 0.92 at V4 / V3–V4 / V5–V7 / whole 16S / RRN, and was the strongest single competitor at the paired amplicon-shotgun species-rank Jaccard, though its false-positive rate at species level remained 10.9 × 10^−4^ higher than Taxonova’s on the digestive V4 mock. SPINGO had species-F medians of 0.58 (V4) and 0.57 (V5–V7) and recovered to ~0.85 on RRN.

Machine learning methods (QIIME 2 naive Bayes, IDTAXA) achieved stronger and more length-stable performance than similarity-based methods (naive Bayes species-F medians 0.74 / 0.80 / 0.87 at V4 / V3–V4 / RRN; IDTAXA 0.67 / 0.75 / 0.93) but trailed Taxonova at every region. The naive Bayes classifier’s independence assumption (each *k*-mer contributes independently to predictions) fails to capture the complex patterns distinguishing closely related species. IDTAXA assigns a bootstrap-supported lineage and accumulates confidence upward through the taxonomy, truncating the lineage where support falls below the threshold; this favours abstention over deep assignment on short amplicons, which limits its species-level recall there.

## Discussion

### Addressing the species-level classification challenge

Species-level taxonomic classification from amplicon sequences has been considered unreliable due to limited phylogenetic resolution in short amplicons [28]. Our results indicate that this limitation partly reflects algorithmic rather than intrinsic information constraints, since several classifiers resolve species that others do not from the same amplicon.

An important distinction merits further emphasis here: Taxonova does not perform amplicon-based phylogenetic species delineation; it performs reference-database species labelling/classification. Hassler *et al.* [29] show that the rRNA gene is too slowly evolving to resolve species de novo for many bacterial lineages, particularly from short hypervariable sub-regions. Greengenes2 sidesteps this by anchoring its species nomenclature in Genome Taxonomy Database (GTDB), which itself uses concatenated core-gene phylogenies and average nucleotide identity (ANI) for species circumscription [30,31]. The 16S rRNA sequence in Greengenes2 is therefore the index into a taxonomy whose biological content is supplied by whole-genome evidence; when Taxonova maps a query 16S to a species label, the label itself is grounded in core-gene phylogeny even though the query is not. For genera with known intragenomic 16S rRNA heterogeneity, recombination, or near-identical sequences across species (*Bacillus cereus sensu lato*, several *Mycobacterium* pairs, *Shigella* / *Escherichia*, parts of Streptomyces) [3], Taxonova’s species call should be interpreted as “the closest reference label” rather than as a systematic identification, and the confidence-aware fallback is designed to abstain at species precisely in this regime.

These species-level results depend on how completely the reference covers the community. The mock communities are closed tests, in which all species are part of the reference, a condition most likely met by human-associated microbiomes, whose species are largely captured by sequenced genomes [56]. In environmental samples such as soil, sludge and natural waters, however, many species are absent from the reference, resulting in over-classification becoming the main species-level error. There, at an under-classification rate of 0.50, IDTAXA over-classified less than Taxonova in the four 16S regions, whereas Taxonova had the lowest over-classification on the full ribosomal operon (S4 Fig; S13 Table). We therefore expect Taxonova’s species-level assignments to be most reliable for human-associated samples, and recommend a higher confidence threshold for environmental samples.

### Confidence-aware classification and handling novel taxa

A strength of Taxonova’s design is the confidence-aware fallback. When species-level evidence is insufficient, returning “genus *X*, species uncertain” is more informative than either forcing an unreliable species assignment or returning no classification at all. The leave-one-clade-out (LOCO) experiment described in Results, “Error modes” (S4 and S5 Tables), tests this behavior directly. Compared at matched abstention, Taxonova reached the lowest mis-classification rate on V3–V4, V5–V7 and RRN, and the lowest over-classification rate on RRN; IDTAXA reached lower over-classification on the four 16S regions. Because abstention on novel sequences is the desired behaviour, the comparison is made at equal under-classification, where the amount of abstention is held fixed and only the quality of the calls that survive it differs. When the true species was removed from the reference, the rank-of-true-taxon (species) confidence dropped sharply while the parent-rank (genus) confidence remained high — producing the user-visible signal “high genus-confidence, low species-confidence” that marks a novel species within a known genus, and the same propagates upward for novel genera within known families. This abstention behaviour may contribute to Taxonova’s lower species-level false-positive rates (a median of 16.2 × 10^−4^ lower than the other classifiers on the mock communities; S1 and S10 Tables); the confidence-threshold sweep underlying this abstention behaviour is shown in S6 Fig.

### Implications for microbiome studies

The reduction in abundance estimation errors (a median of 0.52 to 1.50 in species-level L1 norm, depending on amplicon region) has practical consequences for downstream analyses. Many microbiome studies aim to identify taxa differentially abundant between conditions (e.g., disease versus healthy). Systematic abundance biases from classification errors inflate false positive rates in these analyses, particularly for rare taxa where small absolute errors represent large relative changes. By improving abundance accuracy, Taxonova may increase statistical power while reducing spurious associations.

The strong concordance between Taxonova’s amplicon-derived profiles and shotgun metagenomic references suggests that amplicon sequencing, when coupled with appropriate classification algorithms, can provide reasonable approximations of species-level composition. This does not eliminate the advantages of shotgun metagenomics (strain resolution, functional gene content, absence of primer bias) but demonstrates that amplicon data contains more species-level information than current methods typically extract.

The combination of higher species-level Jaccard with significantly lower Bray–Curtis is the empirical behaviour expected from a classifier that simultaneously detects more of the true species and distributes abundance more accurately across them. The downstream consequences are concrete: reliable recovery of *Faecalibacterium prausnitzii* and *Bacteroides fragilis* in the paediatric IBD cohort [23] matters because both species are mechanistically implicated in mucosal inflammation [32,33]; reliable recovery of *Trebonia kvetii*, *Bradyrhizobium ottawaense / neotropicale*, *Conexibacter woesei*, *Edaphobacter aggregans* and *Paraburkholderia xenovorans* in the Mantri soil panel matters for soil-carbon and N_2_-fixation flux estimates [34,35]; and reliable disambiguation of *Lactobacillus crispatus*, *L. iners*, *L. jensenii*, *Bifidobacterium vaginale* and *Fannyhessea vaginae* in vaginal microbiome surveys matters for bacterial-vaginosis diagnostics and preterm-birth risk stratification [36,37]. These taxa sit precisely at the species level where classifiers either under-resolve or over-commit. More broadly, the principle of trading commit-rate for accuracy at the finest resolvable rank, choosing the deepest rank the signal supports but no deeper, is well established in the structured classification literature [38,39] but has been under-used in microbial taxonomy.

### Limitations

Several limitations warrant consideration:

**Reference-set annotation error.** Public taxonomic references contain non-trivial label noise, empirically ~10% in RDP and ~17% in shared SILVA / Greengenes records [40]. The synthetic-label-noise experiment in S11 Table characterises Taxonova’s robustness as a function of injected error rate (1–20%) at both species and genus levels; Taxonova degrades gracefully and does not cascade catastrophically through ranks. Per-mock species-level F-measure across both noise regimes and injected error rates is shown in S7 Fig.

**16S resolution ceiling.** Even with a perfect classifier and a perfect reference, the 16S gene is too slowly evolving to uniquely resolve every species pair, particularly from sub-region amplicons [29]. For studies where species identity is load bearing (clinical typing, pathogen surveillance, strain tracking) orthogonal evidence (shotgun metagenomics, hybrid-capture, full-length isolate sequencing, MLST) should accompany amplicon-based species calls.

**Amplicon-pipeline pitfalls upstream of classification.** Primer bias, reagent-derived contamination (“kitome”) [41], chimera formation, DNA-extraction biases, 16S copy-number variation and library/sequencing artefacts all distort amplicon surveys upstream of classification and cannot be corrected by classifier choice (see [42] for a comprehensive survey). Taxonova inherits whatever distortion these stages impose and should be paired with standard QC (DADA2 chimera removal [43], decontam [44], PhiX filtering); the 0.02–0.06 species-rank Jaccard between amplicon and shotgun in our real-world panel reflects taxa that PCR primers physically excluded from the library before any classifier was invoked.

**Partial labels.** Reference databases contain many sequences with incomplete taxonomic annotation (e.g., genus but no species label). Taxonova handles partial labels during training but cannot assign species to sequences where no species-level training data exists.

## Conclusions

Taxonova shows that the long-standing difficulty of species-level amplicon classification is, to a certain degree, an algorithmic problem rather than a purely intrinsic limit of the marker. Taxonova is permitted to abstain, returning an informative “genus X, species uncertain” call when the region carries too little signal to separate close relatives. It achieves species-level F-measures of 0.76–1.00 (median 0.94) on short-read and 0.82–0.99 (median 0.97) on long-read amplicons spanning 250 bp to 4,900 bp, while having the lowest species-level false-positive rate of eight classifiers in 38 of the 39 mock-community test sets for which S10 Table gives all eight classifiers, in which every species is in the reference, and reducing species-level abundance-estimation error (L1 norm) by a median of 0.52 to 1.50 depending on amplicon region.

These gains carry through to real data. Against paired shotgun metagenomes from gut, soil and vaginal microbiomes — an independent, primer-bias-free reference — Taxonova achieved the strongest species-level concordance (Jaccard) in all three studies, with a species-level Jaccard index 0.009 to 0.022 higher than that of the next-best classifier. Because the same architecture and abstention rule operate identically across amplicon regions, the improvements are not confined to a single primer set or platform but hold from short hypervariable reads to full ribosomal operons.

The framework is built for routine use: it runs on a single CPU core at roughly 100 sequences per second within a constant 0.82 GB memory footprint, and ships with pre-trained models for the V4, V3–V4, V5–V7, whole 16S and full-ribosomal-operon regions through both a command-line tool and a hosted web application; the accompanying Taxobench framework lets users reproduce the evaluation on their own communities. By combining calibrated abstention with a whole-genome-anchored reference in one classifier, Taxonova converts a recognised bottleneck in amplicon-based microbiome profiling into a practical, deployable capability.

## Materials and methods

### Overview of the classification approach

Taxonova processes sequences through a cascade of seven classifiers, one for each taxonomic level from domain to species (Fig 4a). Each classifier receives (1) the original sequence features, (2) the predictions from all higher taxonomic levels, and (3) learned representations that capture taxonomic context from upstream classifiers. The confidence at each level is bounded by the confidence of the level above. The purpose of this ordering is control over the assignment: because the confidences are nested, a single threshold moves the whole lineage between assigning most sequences and abstaining on nearly all of them, which allows the abstention rate to be set for a given study (S16 Table).

**Fig 4:**
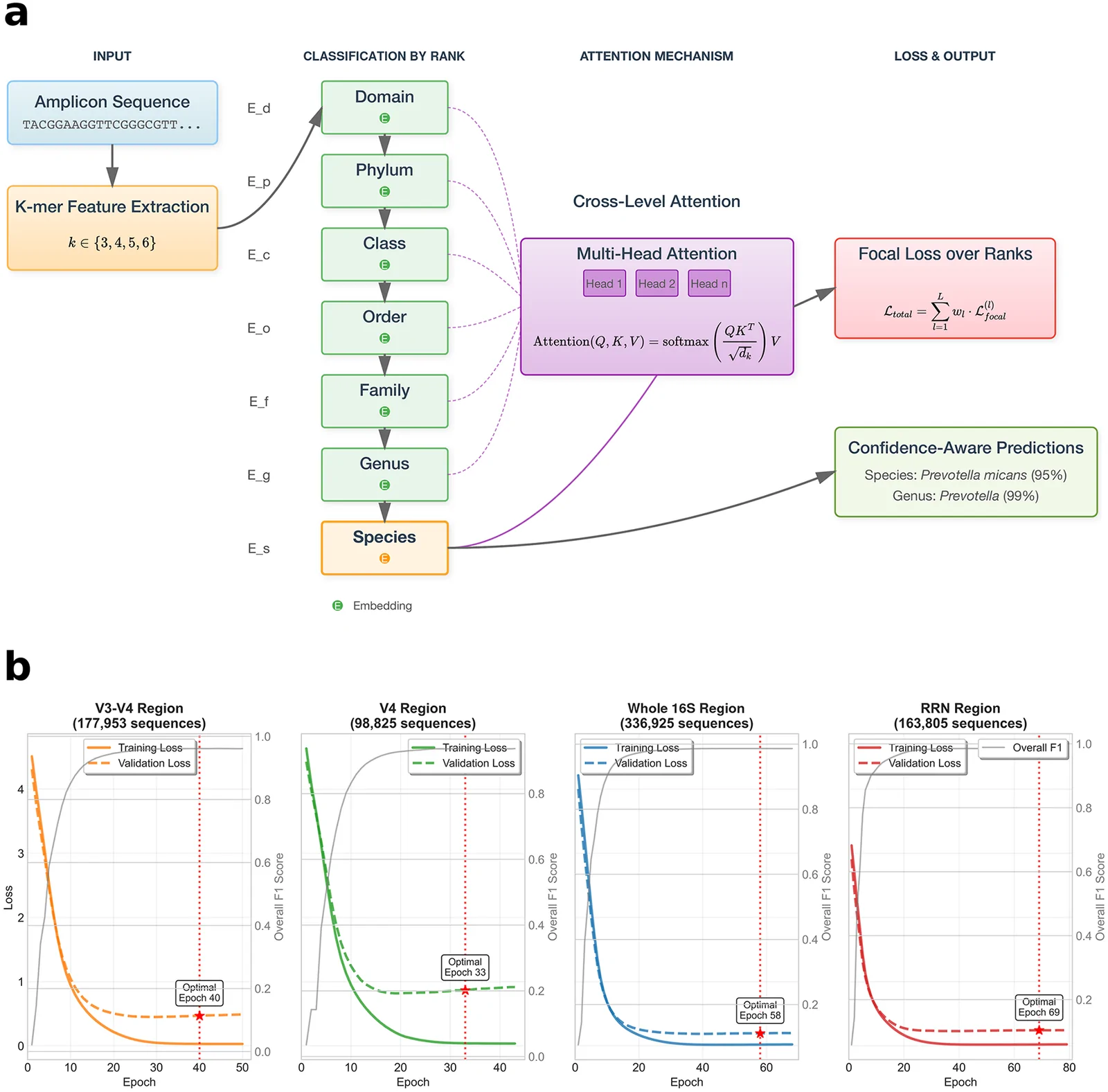
Taxonova methodology and training dynamics. **(a)** Architecture showing how sequence features (represented as *k*-mer frequencies) flow through seven sequential classifiers from domain to species. Each level receives the original features plus embeddings from all preceding classifications, with attention mechanisms enabling selective focus on level-relevant information. **(b)** Training loss curves illustrating convergence behavior during the two-stage training strategy. Stage 1 uses a validation split to determine optimal epochs; Stage 2 retrains on full data for that duration.

### Sequence feature representation

Sequences are represented as normalized frequency vectors of 8-nucleotide *k*-mers. For each input sequence *S*, we count occurrences of all 4,096 possible 8-mers and normalize to probability distributions:

$F_{k}(S)=\left[\frac{c_{\text{1}}}{\sum_{i=\text{1}}^{{\text{4}}^{k}}c_{i}},\frac{c_{\text{2}}}{\sum_{i=\text{1}}^{{\text{4}}^{k}}c_{i}},\ldots ,\frac{c_{{\text{4}}^{k}}}{\sum_{i=\text{1}}^{{\text{4}}^{k}}c_{i}}\right]$

where $c_{i}$ represents the count of the *i*-th *k*-mer. The 6-mer representation balances specificity (capturing phylogenetically informative patterns) with generalization (avoiding overfitting to exact sequence matches). This representation is alignment-free, enabling consistent feature extraction across sequences of different lengths.

### Neural network architecture

**Shared feature processing.** All input *k*-mer features first pass through a shared encoder consisting of layer normalization, two fully connected layers with 512 hidden units each, GELU activation functions, and dropout regularization (rate 0.3). This shared processing extracts general phylogenetic features before specialization at individual taxonomic levels.

**Level-specific classifiers.** Each taxonomic level (phylum through species) has a dedicated classifier that receives a concatenated input comprising: The shared feature representation from the encoder - The predicted class embedding from the preceding level - Accumulated embeddings from all earlier levels

Mathematically, for level l:


$$\begin{array}{c} x_{l}=[h;e_{\text{1}};e_{\text{2}};\ldots ;e_{l-\text{1}}] \end{array}$$

where h is the shared representation, $e_{i}$ is an embedding vector capturing the prediction at level i, and [·;·] denotes concatenation.

**Attention mechanism for cross-level information integration.** Each level-specific classifier incorporates a multi-head attention module (4 heads) that enables selective focus on features from the preceding level. Given features $X_{l}$ at level l and context features $X_{l-\text{1}}$ from the preceding level, the attention computation follows:


$$\begin{array}{c} \text{Attention}(Q,K,V)=\text{softmax}\left(\frac{QK^{T}}{\sqrt{d_{k}}}\right)V \end{array}$$

where queries Q derive from the current level’s features and keys/values K, V derive from the preceding level. This allows, for example, the species classifier to attend to genus-level features that are most informative for distinguishing species within that genus.

### Handling class imbalance with focal loss

Reference databases contain extreme class imbalance: common gut bacteria may have thousands of sequences while rare environmental species have few representatives. Standard cross-entropy loss would cause the model to overfit abundant taxa.

We address this using a focal loss summed over taxonomic levels:


$$\begin{array}{c} L_{total}=\frac{\text{1}}{N_{valid}}\sum_{l=\text{1}}^{\text{7}}w_{l}\cdot L_{focal}^{\left(l\right)} \end{array}$$

where the focal loss for each level is:


$$\begin{array}{c} L_{focal}^{\left(l\right)}=-\alpha_{t}(\text{1}-p_{t}{)}^{\gamma }\text{log}(p_{t}) \end{array}$$

The focal loss parameter γ = 2.5 reduces the loss contribution from well-classified (typically abundant) examples, focusing training on difficult (typically rare) taxa. Class weights $\alpha_{t}$ are dynamically computed using temperature-scaled inverse frequency, updated throughout training to adapt to the observed data distribution. This combination prevents the model from ignoring rare taxa while maintaining accuracy on common taxa.

### Confidence-aware classification

Not all sequences can be confidently classified to species level. Short amplicons may lack sufficient information, or the query may represent a novel species absent from the training database. Rather than forcing unreliable species assignments, Taxonova computes confidence scores at each taxonomic level based on the softmax probability of the predicted class, capped at the confidence of the level above.

When species-level confidence falls below a user-defined threshold (default: 0.8), the classification automatically falls back to the deepest level exceeding its own level threshold. Before the thresholds are applied, the predicted lineage is made consistent with the reference taxonomy by replacing the labels at higher levels with the ancestors of the predicted species. This produces biologically meaningful partial classifications (e.g., “genus *Lactobacillus*, species uncertain”) rather than confident but incorrect species assignments. The confidence threshold can be adjusted for the application: clinical diagnostics may warrant a higher setting for reliability, while exploratory studies may accept a lower one (0.5) for sensitivity. Because a confidence value does not have the same meaning across amplicon regions or reference sizes, we recommend calibrating the threshold to a target abstention rate on a held-out subset of the reference rather than transferring a fixed value between settings. This calibration is available because the confidence at each rank is bounded by the rank above it. In the leave-out analyses, raising the species threshold moved Taxonova from leaving at most 19% of control sequences unassigned to leaving 92% to 100% of them unassigned, depending on the amplicon region. Classifiers trained at each rank alone, on the same reference and with the same training procedures, reached at most 42% at whole 16S and 71% at V5–V7, and could not reach 15 of 100 combinations of region, random seed and pre-specified abstention rate (0.20, 0.30, 0.50 or 0.65) at species rank, compared with 3 of 160 for Taxonova (S16 Table).

### Training strategy

We implemented a two-stage training process to maximize data utilization:

**Stage 1 (Epoch determination):** Using an 80%/20% training/validation split, we train until validation loss stops improving (early stopping with patience of 10 epochs), recording the optimal number of epochs.

**Stage 2 (Full training):** We retrain on 100% of available data for the number of epochs determined in Stage 1, ensuring no data is held out from the final model.

This approach avoids the common trade-off between using data for validation versus training, particularly important for taxonomic classification where rare taxa benefit from every available training example.

### Computational requirements

Taxonova is designed for practical deployment without specialized hardware:

**Inference (classification):** Runs on CPU, processing approximately 100 sequences per second on a standard laptop. Memory usage is under 1 GB regardless of dataset size, as sequences are processed individually. No GPU is used for classification.

**Training:** Benefits from GPU acceleration for the initial model training phase, but pre-trained models for common amplicon regions (V4, V3-V4, V5-V7, whole 16S, and full ribosomal operon) are provided, eliminating the need for users to train models.

**Web interface:** For users who prefer not to install software or lack computational resources, Taxonova is also available as a hosted web application (https://huggingface.co/spaces/claessonlab/Taxonova). The web interface provides the same classification functionality with no installation, no local computational requirements, and immediate access from any web browser. Before classification, the web server screens every submitted sequence with the rRNA gene filter described under “Out-of-distribution input rejection”. This removes all technical barriers to adoption, enabling researchers without bioinformatics expertise to perform species-level taxonomic classification.

### Implementation

Taxonova is implemented in PyTorch 2.x. Training uses `torch.optim.AdamW` with cosine learning-rate schedule, weight decay 1 × 10^−5^, 10% warm-up, batch size 32, and initial learning rate 1 × 10^−2^. Inference is CPU-friendly via `torch.set_num_threads`; GPU (CUDA / Apple-Silicon MPS) is auto-detected and used for training only. The reported architecture uses a *k*-mer length of 8 by default (4^8^ = 65,536 features); the 6-mer configuration mentioned earlier represents one of the values tested during development. Hidden dimension is 512, embedding dimension 64; the cross-level multi-head attention uses 4 heads with dropout 0.3; GELU activations and pre-projection LayerNorm. The focal loss uses γ = 2.5, with α dynamically updated as temperature-scaled inverse class frequency (T = 0.5). Default confidence thresholds are 0.5 for domain through family, 0.7 for genus and 0.8 for species. All seven are user-configurable independently, and when a single confidence value is requested the per-rank thresholds scale with it in this ratio. Source code, conda environments and exact training and inference commands are available at https://github.com/kardokhk/taxonova.

### Reference database

Short-amplicon models (V4, V3–V4, V5–V7, whole 16S) were trained on Greengenes2 2024.09 [6], a unified backbone that places 16S sequences into a whole-genome / core-gene phylogeny built from 380 single-copy marker genes via uDance and imports its taxonomic nomenclature directly from GTDB. Greengenes2 contains 21,074,422 bacterial and archaeal 16S sequences (full-length and partial), making it substantially larger than the legacy Greengenes 13_8 release (frozen in 2013) and the contemporary SILVA 138.1 SSU NR99 reference.

Full-ribosomal-operon (RRN) models were trained on GROND [45], using the build for GTDB release 207 (291,365 16S–ITS–23S operon sequences; https://zenodo.org/records/10889037), from which identical sequences were removed, leaving 163,805 unique sequences. GROND was chosen for the RRN panel because it provides intervening ITS and 23S sequence that SILVA and GTDB 16S extracts do not.

### Benchmarking framework: Taxobench

To systematically evaluate taxonomic classifiers, we developed Taxobench, extending previous benchmarking efforts tax-credit [2] and GSR-DB [46]. Taxobench generates physical-mock and biome-simulated test data with known ground truth, runs eight classifiers through a single harness, and computes standardised performance metrics. Per-classifier output is reformatted to the common Taxobench schema by per-classifier output adapters in the Taxobench repository (src/process_kraken2_output.py, src/process_mapseq_output.py, src/process_idtaxa_output.py, src/process_spingo_output.py and src/process_taxonova_output.py, with QIIME 2-based outputs converted via src/qiime_helper.py; https://github.com/kardokhk/TaxoBench/tree/main/src). The exact CLI invocation, version and parameter set used for every classifier is given in S7 and S8 Tables; gives a compact algorithm-family overview and primary citations.

### Ground-truth construction for simulated communities

MGnify is used as an external source of biome-specific genus-level abundance distributions which seed the composition of each simulated community. The ground-truth taxonomy of every simulated read is the Greengenes2 lineage of the reference sequence the read was simulated from, established by a four-step pipeline: (1) Composition for each biome (digestive system, lung, sputum, soil, skin, mixed, plants), the top-100 genera and their relative abundances are queried from MGnify analyses [22]; only the list of genera and their abundance weights are used downstream — MGnify’s own species-level annotations (which derive from MAPseq + SILVA) are discarded and never enter ground truth or evaluation. (2) Sequence material for each genus, up to 500 reference 16S sequences are drawn from Greengenes2 2024.09, with the per-genus draw size weighted by MGnify abundance. (3) Simulation for short amplicons (V4, V3–V4, V5–V7), ART [47] simulates Illumina reads from the V-region extracted by primer match [48] (5 replicates × 20 reads per feature); DADA2 [43] collapses these reads into ASVs. For long-read regions (whole 16S, RRN), reads are produced by introducing per-base point mutations at rates 0.002–0.02 directly on the reference sequence, ART is not used because its read length is capped at ~300 bp and would silently truncate long amplicons. (4) Ground-truth assignment for ASVs: each DADA2-produced ASV is mapped back to its source reference by gapless alignment (≤ 3 mismatches, reverse-complement aware); matched ASVs inherit the Greengenes2 lineage of their source reference as ground-truth label, and ASVs exceeding the mismatch budget are labelled `other` and contribute to neither the positive nor the negative class.

### Physical mock communities

Three physical mocks with experimentally defined compositions serve as a reference-database-independent cross-check on the biome simulations. Mockrobiota-16S [21] — the union of bacterial 16S mocks that pass two filters (non-NA primer set, no ITS / Eukaryotic primers); the expected composition shipped with each Mockrobiota community is used verbatim as ground truth, and the per-replicate reads are passed through the same primer-trim and DADA2 ASV-inference pipeline as the biome mocks. gutmock — a high-fidelity PacBio HiFi full-length 16S mock of the canonical paediatric-gut microbiome, constructed in-house from circular consensus sequencing (CCS) reads of reference isolates; its 339 source sequences all belong to the genus *Prevotella* in the Greengenes2 taxonomy and carry 81 species labels. Full-length reads are sub-sampled to V4, V3–V4 and whole 16S by primer match. For V5–V7, the region was extracted from the full-length sequences by primer match, Illumina reads were simulated with ART and denoised with DADA2 as for the biome-simulated communities, and each sequence variant was given the ground-truth label of the source sequence it matches; variants that match more than one source species equally well keep a genus-level label. vaginalmock — a companion full-length 16S mock of the canonical vaginal microbiome built by the same in-house procedure from Lactobacillus-dominant references plus the canonical BV-associated taxa (*Bifidobacterium vaginale /* formerly Gardnerella vaginalis, Fannyhessea vaginae, Megasphaera lornae*); V4, V3–V4, V5–V7 and whole 16S are evaluated, and the V5–V7 test set was produced by extraction, ART simulation and DADA2 as for gutmock.

### TAXXI benchmark

The TAXXI benchmark [55] provides each dataset split into two halves, which are used in turn for training and for testing, together with name counts that define which test species are present in the training half. We trained a Taxonova model on each training half and classified the corresponding test half without applying any confidence threshold, so that thresholds could be applied afterwards to the output of a single classification run. Because training sets of 1,000–4,000 sequences are too small for the epoch schedule used with Greengenes2, training was limited to 150 epochs with early stopping, and the number of epochs reached was recorded for each run.

The metrics follow the definitions in the TAXXI source code. Test sequences whose species occurs in the training half (known sequences) were scored for true positives, false negatives and mis-classification, and the other test sequences (novel sequences) for true negatives and over-classification. Each rate was averaged over the two directions of the cross-validation, and accuracy was calculated as the number of correct assignments divided by the number of known plus over-classified sequences. As in the benchmark, combinations with fewer than 10 known sequences were not reported. In each comparison, Taxonova was evaluated at the threshold whose under-classification rate (the false-negative rate over known sequences) was closest to that of the other classifier. For S12 Fig, Taxonova’s over-classification, mis-classification and under-classification rates were calculated at every threshold from 0 to 1 in steps of 0.0001, with the same threshold applied to all ranks; in these datasets the thresholds used throughout this study select the same species-level assignments as a single threshold of 0.7.

The benchmark provides summary results for the other classifiers but not their assignment of each test sequence. Our scoring script follows the published source code and also reports the numbers of known and novel sequences, which do not depend on the classifier.

### Evaluation metrics

We computed standard classification metrics at each taxonomic level: Precision *P* = *TP* / (*TP* + *FP*); Recall *R* = *TP* / (*TP* + *FN*); F-measure *F* = 2 *PR* / (*P* + *R*); and L1 / L2 abundance error L_1_ = Σ |pᵢ − p̂ᵢ|, L_2_ = (Σ (pᵢ − p̂ᵢ)²)^½. Predictions emitted as species-group brackets `[a | b | c]` by Taxonova are evaluated bracket-aware: if any bracket member matches the truth species, the ASV is credited as a true positive (with abundance split equally if multiple members match); if no member matches, the bracket is counted as a single false positive (not as N false positives). All other classifiers’ predictions pass through unchanged. The bracket-aware evaluator and the default-evaluator paths are bit-identical for bracket-less predictions.

### Paired amplicon-shotgun studies

**Amplicon preprocessing.** Primers were removed with cutadapt v4 (default error rate 0.1). Reads were quality-filtered and denoised with DADA2 v1.30 (`filterAndTrim` with `maxEE=(2, 2)`, `truncQ = 2`, `truncLen = 230/200` for V4 forward / reverse and `280/220` for V3–V4). ASVs supported by ≥ 2 reads were retained.

**Shotgun preprocessing.** Adapter-trimming was performed with fastp v0.23; host-read removal with bowtie2 v2.4 [49] against GRCh38 (for the human studies) was followed by alignment to the Web of Life 2 (WoL2) reference [50] with bowtie2 `--very-sensitive-local`; taxonomic profiling used Woltka [51] with Greengenes2 taxonomy (`woltka classify` default).

**Concordance metrics.** For each (study, classifier, paired-sample) we compute per-sample Jaccard and Bray–Curtis at species rank from two relative-abundance vectors: the amplicon ASV table folded through the classifier’s prediction, and the paired-shotgun OGU table folded through Greengenes2 taxonomy. Per-study aggregate statistics are means over paired samples; per-classifier statistical significance is assessed by paired Wilcoxon signed-rank tests over per-sample values. Jaccard and Bray–Curtis are reported jointly because the two metrics capture complementary information: Jaccard scales with the size of the species intersection and remains discriminating at low overlap, while Bray–Curtis captures abundance concordance and can saturate into a statistically indistinguishable band when the absolute species-overlap fraction is small (≤ 10%), as is the case across all eight classifiers on every study in this panel — a property of the underlying biology of amplicon-vs-shotgun comparison rather than of any classifier choice.

Code for amplicon preprocessing, shotgun preprocessing and concordance metrics in this section is available in the Taxobench repository (https://github.com/kardokhk/TaxoBench): the amplicon (primer trimming and DADA2 denoising) and shotgun (bowtie2 alignment and Woltka profiling) preprocessing recipes are documented in docs/realworld.md and docs/realworld_amplicon_qc.md, and the per-sample concordance metrics are computed in scripts/05_realworld_evaluate.py.

### Mis-classification vs. over-classification

Mock communities cannot measure over-classification, because all of their taxa are in the reference [2,13]. Following the evaluation of IDTAXA [13], we therefore built for each region a leave-one-clade-out reference by removing a stratified selection of 200 species, 100 genera, 50 families, 25 orders and 10 classes, and a second reference from which 2,000 species were removed, with three independent random selections of species. A species was not removed if its removal would leave its genus with fewer than four sequences; at V4, therefore, only 1,817 species could be removed, and the three selections were identical. Removing 2,000 species also removed 17–33% of the reference sequences, so the two analyses differ both in the number of removed species and in reference size.

All eight classifiers were retrained, or had their reference index rebuilt, on each reduced reference. Each classifier then classified the sequences of the removed taxa (novel sequences) and a control set of 2,000 held-out sequences whose taxa remained in the reference. All eight report a graded confidence, so each was evaluated over a range of confidence thresholds. For each rank *L* and confidence threshold *τ*, we calculated the MC rate as the fraction of control sequences with a confident but wrong assignment at rank *L*; the OC rate as the fraction of novel sequences whose taxon at rank *L* was removed that nevertheless received a confident assignment at rank *L*; and the under-classification (UC) rate as the fraction of control sequences with no assignment at rank *L*. Because a confidence value does not have the same meaning in different classifiers, or in the same classifier in different amplicon regions, MC and OC were compared at the same UC rate rather than at the same threshold, following Murali *et al.* [13]; the threshold is the parameter along each curve and is not plotted (S4 Fig).

The thresholds were placed at quantiles of the control sequences’ confidence at their least confident rank, so that they are spread evenly along the UC axis. Thresholds at equal intervals would not be: the median of this confidence is 0.26 at V5–V7 but 0.999 on the rRNA operon, where steps of 0.01 would cross a UC rate of 0.5 in a single step. With quantile placement, the OC rate at a given UC rate is determined to within 0.005 in every region, and the UC rate actually reached is reported with every value (S4, S5 and S13 Tables). BLASTn, VSEARCH, Kraken 2 and SPINGO produce only 26, 16, 17 and 101 distinct species-level confidence values and were evaluated at the UC rates these allow, without interpolation.

For the comparison between classifiers, Taxonova’s seven thresholds were set to the same value; the default per-rank thresholds are given under “Confidence-aware classification”. VSEARCH was run through QIIME 2 with `--p-maxrejects 32` in the leave-out analyses, because the exhaustive QIIME 2 default was projected to take 171 h at whole 16S and 1,830 h on the rRNA operon for a single reference. At V5–V7, this setting gave the same genus and species assignments as the exhaustive search for all 2,000 control sequences, and 72 more species assignments among 103,242 novel sequences (0.07%), so it may slightly overestimate VSEARCH’s over-classification.

### Confidence-threshold sweep

Because the eight evaluated classifiers use non-comparable confidence semantics (Taxonova: per-rank softmax posterior; naive Bayes: posterior under the multinomial NB; IDTAXA: a bootstrap-derived per-rank score; SPINGO: bootstrap support fraction; VSEARCH / BLAST: consensus fraction over top-k hits; Kraken 2: fraction of *k*-mers under the LCA; MAPseq: a per-rank combined alignment + *k*-mer score), the main-text point estimates are read at matched under-classification rather than at a matched threshold, and each tool’s published default is reported separately (S4 Table). We additionally provide a confidence-threshold sweep (S6 Fig) covering Mockrobiota-16S, and digestive system biome across V4, V3–V4 and V5–V7 — for per-rank-confidence tools the sweep is post-hoc (one permissive run + 21-point recut), for single-confidence tools (naive Bayes, VSEARCH, BLAST) it is run-time at 4–5 native settings. Curves are informative within tool and only loosely comparable across tools.

### Label-noise robustness

Public taxonomic references contain non-trivial annotation error [40]. We characterised Taxonova’s robustness by injecting two classes of synthetic noise into the V4 training reference (species-only mis-labelling within the same genus; full genus-misplacement within the same family) at rates *r* ∈ {0, 1, 5, 10, 20} %, retraining at each level, and evaluating on the held-out clean mock panel. Results are in S11 Table.

### Out-of-distribution input rejection

Taxonova was trained exclusively on 16S rRNA gene sequences (Greengenes2) and 16S–ITS–23S operons (GROND). It does not have an internal out-of-distribution detector; on non-rRNA inputs (protein-coding genes, random nucleotides, tandem repeats) its softmax output is unreliable, as is the case for any supervised neural classifier without explicit OOD handling [52]. We therefore created an upstream HMM-based sequence guard that screens every input FASTA with barrnap-bac (HMMER-based bacterial rRNA gene finder) and Infernal `cmscan` against the Rfam SSU rRNA covariance models at *E*-value ≤ 1 × 10^−3^. A sequence passes the guard if either screen returns a hit; otherwise, the wrapper emits a row with `Unclassified` at every rank, without ever invoking the classifier. Surviving sequences are passed unchanged to the production Taxonova checkpoint. On a 1,000-sequence V4 adversarial panel (coding sequence, random nucleotides, shuffled 16S, tandem repeats at 250 bp), the prefilter rejected 100% of inputs at every rank — closing a 9.2% phylum-level “confident leak” (at confidence ≥ 0.9) that is otherwise observed on the same panel. On the legitimate V4 mock panel, prefilter activation produces a species-F delta of 0.000 on every cell; on the V4 real-world panel, per-study |ΔJaccard| ≤ 0.004 and |ΔBC| ≤ 0.018, within run-to-run noise.

### Compared methods

We evaluated Taxonova against seven published classifiers representing the major algorithmic approaches in current use (S8 Table):

**Similarity-based methods:** BLASTn [9]: Sequence alignment with consensus taxonomy from top hits (97% identity threshold, majority consensus from top 10 matches) - VSEARCH [10]: Fast sequence alignment similar to BLASTn (97% identity threshold)

***K*-mer and probabilistic methods:** Kraken 2 [14]: Exact *k*-mer matching against a reference database; originally designed for metagenomic reads rather than amplicons - MAPseq [53]: *K*-mer matching with confidence estimation based on database coverage - SPINGO [54]: *K*-mer based classification with bootstrap confidence estimates

**Machine learning methods:** QIIME 2 naive Bayes [2]: Multinomial naive Bayes classifier trained on *k*-mer frequencies; the default classifier in QIIME 2 and the basis for Mothur and RDP-Classifier implementations - IDTAXA [13]: Combines distance-based and machine-learning classification, assigning a bootstrap-supported lineage and accumulating confidence upward through the taxonomy

All classifiers were run with the default parameters given in their documentation, with references derived from Greengenes2 for V4, V3–V4, V5–V7 and whole 16S and from GROND for RRN, in the form described in the next section. In the mock community and paired shotgun analyses and in the TAXXI benchmark, Taxonova was run with confidence thresholds of 0.5 for domain to genus and 0.7 for species, not with the software’s default species threshold of 0.8; in the leave-out analyses, thresholds were applied after classification (see “Mis-classification vs. over-classification”). S7 Table gives the version, command, key parameters and reference database for each classifier.

### Reference sets supplied to each classifier

Two kinds of reference were used, for different purposes.

In the leave-out analyses, all eight classifiers were built from the same reduced reference in each region, supplied as the same sequence and taxonomy files (`sequences.fasta` and `taxonomy.tsv`). After removal of the held-out taxa, these references contained 83,912 sequences at V4, 155,456 at V3–V4, 275,200 at V5–V7, 256,406 at whole 16S and 122,981 at RRN (S14 Table). All results of the leave-out analyses are based on these references.

In the mock community and paired shotgun analyses, each classifier used the reference in the form that its authors distribute or recommend, so the references differ between classifiers. IDTAXA, QIIME 2, SPINGO and MAPseq used the full-length reference even for short amplicons, as they are designed to be used. Taxonova used a reference trimmed to the amplicon region, because it trains a separate model for each region. BLASTn and VSEARCH used the complete reference for the amplicon region, including sequences without a species name, because both assign taxonomy by consensus over their best hits and a reference restricted to sequences with a species name would bias this consensus towards species-level assignments. S14 Table gives the number of sequences supplied to each classifier in each region. These analyses therefore evaluate the classifiers as they are normally used.

In this study, the amplicon of about 1,500 bp that spans the complete 16S rRNA gene is called whole 16S. The software and its configuration files name this region `V1-V9`, after the V1 forward and V9 reverse primers that generate it. The full ribosomal operon (16S–ITS–23S, about 4,900 bp) is called RRN.

### Ethics statement

This study utilized only publicly available data from previously published studies. No ethics approval was required.

## Supporting information

S1 Table Full per-classifier metrics for the biome-simulated short-amplicon mocks (digestive system, lung, sputum, skin, soil, mixed, plants) and the seven matched gtdb full-operon mocks. Long-form table with one row per (mock × region × classifier × rank) reporting bracket-aware precision, recall, F-measure, specificity, L1-norm and L2-norm abundance errors at five ranks (Class through Species).
(CSV)

S2 Table Detailed metrics for the two in-house physical mock communities (gutmock and vaginalmock) across V4, V3–V4 and whole 16S, and V5–V7 for gutmock (vaginalmock at V5–V7 is given in Table 1) — per-rank precision, recall, F-measure, specificity, L1 and L2. (CSV)

S3 Table V5–V7 detailed metrics for Mockrobiota-16S, gutmock and the seven biome-simulated mocks (vaginalmock at V5–V7 is given in Table 1). Per-rank precision, recall, F-measure, specificity, L1 and L2 across eight classifiers.
(CSV)

S4 Table LOCO mis-classification (MC) and over-classification (OC) rates at each classifier’s published default confidence threshold τ (for Taxonova, 0.7 at the rank evaluated), per region × classifier × rank. Includes the underlying held-in / held-out counts (n_mc_total, n_mc_errors, n_mc_abstain, n_oc_total, n_oc_errors, n_oc_abstain) and the abstention rates. All eight classifiers (Taxonova, IDTAXA, QIIME 2, BLAST consensus, VSEARCH, SPINGO, Kraken 2 and MAPseq); each reports a graded confidence and is read at its own published default.
(CSV)

S5 Table LOCO MC / OC full confidence-threshold sweep — long-form companion to S4 Table. Per (region × classifier × rank × τ) MC and OC across the swept thresholds, which are placed at empirical quantiles of each region’s weakest-rank control confidence rather than on a uniform ladder (see Methods), enabling the full MC-versus-UC trade-off curve at all five ranks for every (region, classifier) pair.
(CSV)

S6 Table L1-norm and L2-norm abundance error per (mock × region × classifier × rank) across the full trimmed evaluation panel (3 physical mocks + 7 biome mocks + 7 gtdb RRN mocks). (CSV)

S7 Table Per-classifier command-line invocations, software versions and parameter values used in the benchmark. For each of the eight evaluated classifiers (Taxonova, BLASTn, VSEARCH, Kraken 2, MAPseq, SPINGO, IDTAXA, QIIME 2 naive Bayes) the table reports tool name, version, exact default command line, and the parameter combination from configs/classifiers_default.
(CSV)

S8 Table Compact classifier method overview: algorithm family, hierarchical flag, reference database used at short and long amplicon length, and primary literature citation, for each of the eight evaluated classifiers. Companion to S7 Table.
(CSV)

S9 Table Per-sample paired amplicon–shotgun metrics for the three real-world studies — Zuo et al. 2022 (42 paediatric gut V4 samples), Mantri et al. 2020 (7 paired soil V4 samples) and Virtanen et al. 2019 (21 paired vaginal V3–V4 samples). Long-form table with one row per (study × classifier × rank × paired sample) reporting Jaccard index, Bray–Curtis distance, Aitchison distance, precision, recall, F-measure, L1, L2 and per-sample taxa counts.
(CSV)

S10 Table Specificity and detection rate per (mock × region × classifier × rank) across the full panel. Bracket-aware metrics from the BRACKETFIX evaluator.
(CSV)

S11 Table Label-noise robustness V4 retrains. Per (noise_regime × noise_rate × mock × rank) TP / FP / FN / precision / recall / F-measure for V4 Taxonova retrains at synthetic training-label noise rates r ∈ {0, 1, 5, 10, 20} % under two noise regimes (species_within_genus: random species swaps within the same genus; genus_within_family: random species + genus swaps within the same family). All retrains evaluated on the seven clean V4 biome mocks at species and genus ranks.
(CSV)

S12 Table HMM-prefilter adversarial panel and legitimate-input delta. Per (adversary_class) catch rate of the optional barrnap-bac + Infernal/Rfam SSU covariance-model 16S prefilter on the 1000-sequence V4 adversarial panel (cds, random, shuffled_16s, tandem at 250 bp), together with the residual confident-species and confident-phylum leak rates at the standard thresholds and the headline cost on legitimate inputs (Δ species-F = 0.000 on the V4 mock communities; |Δ Jaccard| ≤ 0.0037 and |Δ Bray–Curtis| ≤ 0.018 in the V4 paired shotgun studies).
(CSV)

S13 Table Species-level error rates in the leave-out analysis with 2,000 species removed, and the under-classification needed to keep over-classification below 5%. (a) Over-classification and mis-classification of all eight classifiers in each region, at the evaluated threshold whose under-classification rate on the control sequences is closest to 0.50 and to 0.65, with the rate actually reached and the numbers of control and novel sequences. (b) For each classifier and region, the lowest under-classification rate at which species-level over-classification falls below 0.05, and the highest of these values across regions; `never` means that no threshold reaches this limit. Kraken 2 reaches the limit in every region only at an under-classification rate of 1.000, that is, by leaving every sequence unassigned. (c) Taxonova’s species-level over-classification at under-classification rates of 0.50 and 0.65 for each of the three random selections of removed species, with the size of the remaining reference and the number of novel sequences, because the selections remove different amounts of the reference. V4 is not included in (c), because only 1,817 of its species could be removed, so the three selections were identical.
(XLSX)

S14 Table Reference sequence set supplied to each classifier, per analysis. (a) The leave-out analysis, in which every classifier is built from the identical held-out reference in every region, verified by sequence count; all error-rate and novel-taxon results rest on this. (b) The mock-community and paired-shotgun analyses, in which each classifier receives the reference its authors distribute, with the sequence count given per classifier and region. (c) Classifiers whose reference is not directly sequence-countable, recorded as text.
(XLSX)

S15 Table TAXXI benchmark at species rank: all 133 comparisons of Taxonova with the seven other classifiers in the 19 combinations of dataset and identity level. Each row gives the accuracy and the mis-classification (MCR), under-classification (FNR) and over-classification (OCR) rates of the other classifier, as published with the benchmark, and of Taxonova at the threshold whose under-classification rate is closest to that of the other classifier. The columns beginning taxonova_default give the same values at the thresholds used throughout this study (0.5 for domain to genus, 0.7 for species), with Taxonova’s rank among the eight classifiers by species accuracy (taxonova_default_rank_tied marks ties). n_known_queries and n_novel_queries are the numbers of test sequences whose species is present in and absent from the reference; n_known_queries is 177–6,097 in the seven combinations at 99% identity and 10–122 in the other twelve.
(CSV)

S16 Table Range of assignment behaviour available to Taxonova and to classifiers trained at each rank alone. For each amplicon region and for species and genus rank, the highest under-classification rate each model reached when its confidence threshold was raised to the maximum, that is, the largest share of control sequences in the leave-out analysis that it could be made to leave unassigned. Classifiers trained at each rank alone are seven PyTorch networks, one per rank, trained on the same reference and for the same number of epochs as Taxonova, in two versions: with cross-entropy loss only, and with the training procedures used for Taxonova. Five random seeds for each version and eight for Taxonova. Differences are Taxonova minus the per-rank classifiers, with 95% confidence intervals and *P* values from Welch *t* tests across seeds. The final rows give, for each model and rank, how many combinations of region, seed and target under-classification rate (0.20, 0.30, 0.50, 0.65) could not be reached, above the highest or below the lowest reachable rate.
(CSV)

S1 Fig Real-world species-level community composition, gut and vaginal. Stacked relative-abundance bars of the species recovered by each classifier compared with the paired shotgun reference, for (a) Zuo et al. (paediatric gut, V4) and (b) Virtanen et al. (vaginal, V3–V4). Taxa below 1% relative abundance are pooled into an “Other” category. The soil study is in S2 Fig.
(TIF)

S2 Fig Real-world species-level community composition, soil. As S1 Fig, for Mantri et al. (soil, V4).
(TIF)

S3 Fig Real-world species-level Bray–Curtis concordance, ranked. Per-study species-rank Bray–Curtis distance between amplicon-derived and paired shotgun profiles, with classifiers ranked within each study (lower is better) for Zuo et al., Virtanen et al. and Mantri et al. Companion to the Jaccard panel (Fig 1e).
(TIF)

S4 Fig Leave-one-clade-out error curves at species rank. Over-classification (top row: the fraction of novel sequences that received a confident species-level assignment) and mis-classification (bottom row: the fraction of control sequences that received a confident but wrong species-level assignment), plotted against the under-classification rate for each amplicon region and all eight classifiers. The confidence threshold is the parameter along each curve and is not plotted, so the classifiers are compared at the same under-classification rate rather than at the same threshold. BLASTn, VSEARCH, Kraken 2 and SPINGO produce only 26, 16, 17 and 101 distinct species-level confidence values, so their curves are step functions. The values at each classifier’s published default are in S4 Table and all values in S5 Table; the curves at genus, family, order and class rank are in S8–S11 Figs.
(TIF)

S5 Fig Abundance-estimation L1-norm error. Per-rank species-level L1-norm error (sum of absolute differences between observed and expected relative abundances; range 0–2, lower is better) for (a) the three physical mock communities and (b) the seven biome-simulated mocks plus the matched full-operon (RRN) sets, each cell averaged over its panel. Companion to the L2-norm heatmaps in Fig 1c, 1d; Taxonova (leftmost column) shows the smallest error in every region.
(TIF)

S6 Fig Confidence-threshold sweep. Species-level F-measure of all eight classifiers as a function of the confidence threshold on Mockrobiota-16S (top row) and the digestive-system biome mock community (bottom row), at V3–V4, V4 and V5–V7 (columns). Because the classifiers compute confidence differently, the curves can be compared within a classifier but only loosely between classifiers (see Methods).
(TIF)

S7 Fig Label-noise robustness. Per-mock species-level F-measure of V4 Taxonova retrains under injected reference label noise (1–20%) for both noise regimes (species labels exchanged within a genus, and genus labels exchanged within a family). Tabulated in S11 Table.
(TIF)

S8 Fig Leave-one-clade-out error curves at genus rank. As S4 Fig, at genus rank.
(TIF)

S9 Fig Leave-one-clade-out error curves at family rank. As S4 Fig, at family rank.
(TIF)

S10 Fig Leave-one-clade-out error curves at order rank. As S4 Fig, at order rank.
(TIF)

S11 Fig Leave-one-clade-out error curves at class rank. As S4 Fig, at class rank.
(TIF)

S12 Fig Over-classification and mis-classification against under-classification on the TAXXI benchmark. Species-level over-classification rate (a–c: the percentage of test sequences whose species is absent from the training set that received a species-level assignment) and mis-classification rate (d–f: the percentage of test sequences whose species is present in the training set that received a wrong species), plotted against the under-classification rate (the percentage of test sequences whose species is present in the training set that received no species-level assignment), for the three full-length datasets of the benchmark at 99% identity. BLAST16S, BLAST16S/10 and WITS are the benchmark’s names for the datasets ncbi_16s, ten_16s and rdp_its of S15 Table, which also gives their V4 and V3–V5 subsets. Each panel title gives the numbers of test sequences whose species is present in the training set (known) and absent from it (novel). The line shows Taxonova at every confidence threshold, with the same threshold applied to all ranks, and the open square its operating point at the thresholds used throughout this study. The other symbols show the seven classifiers evaluated in the benchmark, at the values published with it [55] and named as in the benchmark, with the codes of S15 Table in parentheses. Each curve and each point is the mean of the two directions of the cross-validation.
(TIF)
